# Development of a Panel of Genome-Wide Ancestry Informative Markers to Study Admixture Throughout the Americas

**DOI:** 10.1371/journal.pgen.1002554

**Published:** 2012-03-08

**Authors:** Joshua Mark Galanter, Juan Carlos Fernandez-Lopez, Christopher R. Gignoux, Jill Barnholtz-Sloan, Ceres Fernandez-Rozadilla, Marc Via, Alfredo Hidalgo-Miranda, Alejandra V. Contreras, Laura Uribe Figueroa, Paola Raska, Gerardo Jimenez-Sanchez, Irma Silva Zolezzi, Maria Torres, Clara Ruiz Ponte, Yarimar Ruiz, Antonio Salas, Elizabeth Nguyen, Celeste Eng, Lisbeth Borjas, William Zabala, Guillermo Barreto, Fernando Rondón González, Adriana Ibarra, Patricia Taboada, Liliana Porras, Fabián Moreno, Abigail Bigham, Gerardo Gutierrez, Tom Brutsaert, Fabiola León-Velarde, Lorna G. Moore, Enrique Vargas, Miguel Cruz, Jorge Escobedo, José Rodriguez-Santana, William Rodriguez-Cintrón, Rocio Chapela, Jean G. Ford, Carlos Bustamante, Daniela Seminara, Mark Shriver, Elad Ziv, Esteban Gonzalez Burchard, Robert Haile, Esteban Parra, Angel Carracedo, for the LACE Consortium

**Affiliations:** 1 University of California San Francisco, San Francisco, California, United States of America; 2 Instituto Nacional de Medicina Genómica, Mexico City, Mexico; 3 Case Western Reserve University, Cleveland, Ohio, United States of America; 4 Fundación Pública Galega de Medicina Xenómica (SERGAS)-CIBERER, Universidade de Santiago de Compostela, Santiago de Compostela, Spain; 5 Universitat de Barcelona, Barcelona, Spain; 6 Universidad del Zulia, Maracaibo, Venezuela; 7 Universidad del Valle, Santiago de Cali, Colombia; 8 Universidad Industrial de Santander, Bucaramanga, Colombia; 9 Universidad de Antioquia, Medellín, Colombia; 10 Instituto de Investigaciones Forenses, Sucre, Bolivia; 11 Universidad Tecnológica de Pereira, Pereira, Colombia; 12 Unidad de Genética Forense, Servicio Médico-Legal de Chile, Santiago de Chile, Chile; 13 University of Michigan, Ann Arbor, Michigan, United States of America; 14 University of Colorado at Boulder, Boulder, Colorado, United States of America; 15 Syracuse University, Syracuse, New York, United States of America; 16 Universidad Peruana Cayetano Heredia, Lima, Peru; 17 Wake Forest University, Winston-Salem, North Carolina, United States of America; 18 Universidad Mayor de San Andrés, La Paz, Bolivia; 19 Centro Medico Nacional Siglo XXI, Mexican Social Security Institute (IMSS), Mexico City, Mexico; 20 Hospital General Regional 1, IMSS, Mexico City, Mexico; 21 Centro de Neumología Pediátrica, San Juan, Puerto Rico; 22 VA Caribbean Health System, San Juan, Puerto Rico; 23 Instituto Nacional de Enfermedades Respiratorias (INER), Mexico City, Mexico; 24 Johns Hopkins Bloomberg School of Public Health, Baltimore, Maryland, United States of America; 25 Stanford University, Stanford, California, United States of America; 26 National Cancer Institute, Bethesda, Maryland, United States of America; 27 Penn State University, University Park, Pennsylvania, United States of America; 28 University of Southern California, Los Angeles, California, United States of America; 29 University of Toronto at Mississauga, Mississauga, Canada; Georgia Institute of Technology, United States of America

## Abstract

Most individuals throughout the Americas are admixed descendants of Native American, European, and African ancestors. Complex historical factors have resulted in varying proportions of ancestral contributions between individuals within and among ethnic groups. We developed a panel of 446 ancestry informative markers (AIMs) optimized to estimate ancestral proportions in individuals and populations throughout Latin America. We used genome-wide data from 953 individuals from diverse African, European, and Native American populations to select AIMs optimized for each of the three main continental populations that form the basis of modern Latin American populations. We selected markers on the basis of locus-specific branch length to be informative, well distributed throughout the genome, capable of being genotyped on widely available commercial platforms, and applicable throughout the Americas by minimizing within-continent heterogeneity. We then validated the panel in samples from four admixed populations by comparing ancestry estimates based on the AIMs panel to estimates based on genome-wide association study (GWAS) data. The panel provided balanced discriminatory power among the three ancestral populations and accurate estimates of individual ancestry proportions (R^2^>0.9 for ancestral components with significant between-subject variance). Finally, we genotyped samples from 18 populations from Latin America using the AIMs panel and estimated variability in ancestry within and between these populations. This panel and its reference genotype information will be useful resources to explore population history of admixture in Latin America and to correct for the potential effects of population stratification in admixed samples in the region.

## Author Summary

Individuals from Latin America are descendants of multiple ancestral populations, primarily Native American, European, and African ancestors. The relative proportions of these ancestries can be estimated using genetic markers, known as ancestry informative markers (AIMs), whose allele frequency varies between the ancestral groups. Once determined, these ancestral proportions can be correlated with normal phenotypes, can be associated with disease, can be used to control for confounding due to population stratification, or can inform on the history of admixture in a population. In this study, we identified a panel of AIMs relevant to Latin American populations, validated the panel by comparing estimates of ancestry using the panel to ancestry determined from genome-wide data, and tested the panel in a diverse set of populations from the Americas. The panel of AIMs produces ancestry estimates that are highly accurate and appropriately controlled for population stratification, and it was used to genotype 18 populations from throughout Latin America. We have made the panel of AIMs available to any researcher interested in estimating ancestral proportions for populations from the Americas.

## Introduction

Most individuals from the Americas are admixed descendants of Native American, European, and African ancestors. Complex historical factors have resulted in varying proportions of ancestral contributions between individuals within and between ethnic groups [1]. For example, in a study of five Hispanic/Latino ethnic groups, Puerto Ricans and Dominicans showed the largest proportion of African ancestry, while Mexicans had a significantly larger proportion of Native American ancestry than the other groups [2]. Even within small islands in the Caribbean there can be high variance in admixture proportions [3]. Ancestry Informative Markers (AIMs) are commonly used to estimate overall admixture proportions efficiently and inexpensively [4]. AIMs are polymorphisms that exhibit large allele frequency differences between populations and can be used to infer individuals' geographic origins. For example, the forensic use of a panel of AIMs successfully identified the ancestral origin of seven unmatched samples implicated in the 11-M Madrid commuter train bombings of 2004 [5]. Using a panel of AIMs distributed throughout the genome, it is possible to estimate the relative ancestral proportions in admixed individuals such as African Americans and Latin Americans, as well as to infer the time since the admixture process [6], [7].

In addition to providing estimates of individual's ancestral history, admixture proportions can be correlated to physiologic measurements such as spirometric measurements of lung function [8] and uterine artery blood flow [9], risk of diseases such as peripheral vascular disease [10] and breast cancer [11], as well as to control for the effects of population stratification in genetic association studies [12]. Consequently, it is important for researchers to have access to validated, accurate panels of AIMs that can be used for Latin American populations throughout the Americas, including Hispanics/Latinos in the United States, where according to the US census bureau, they are the fastest growing ethnic group [13].

Several groups have described panels of AIMs designed to estimate individual ancestry and to control for the effects of population stratification in Latino populations [14], [15], [16], [17]. However, in most cases these studies were limited in the number of AIMs selected, lack of systematic basis for the selection of AIMs, and lack of validation compared to robust estimates of ancestry based on genome-wide data from hundreds of thousands of markers. Additionally, most published AIMs panels lack availability of genotyping data of relevant ancestral populations.

In this paper, we describe a three-stage approach to developing a panel of 446 Ancestry Informative Markers (AIMs) optimized to characterize admixture throughout Latin America. In the first stage, we used genome-wide data from two African populations, three European populations, and six Native American populations to select AIMs that were informative, evenly distributed throughout the genome, and portable, having little within-continent heterogeneity. In the second stage, we validated the panel of AIMs in four admixed samples by comparing the ancestry estimates based on the AIMs panel with ancestry estimates based on genome-wide data. In the final stage, using these AIMs, we genotyped samples from 18 additional populations originating throughout the Americas to estimate ancestry differences within and between populations and to determine the onset of admixture for each group.

## Results

### AIMs selection

A total of 446 AIMs were identified; the panel is presented in its entirety in Table S1. The 400 most informative markers were used to design multiplexes for the Sequenom genotyping platform. Consistent with the goals of the study, the AIMs panel provides a balanced set of markers capable of distinguishing the three ancestral populations of modern Latin Americans. Specifically, the cumulative locus-specific branch length for the *I_n_* statistic was 43.8, 44.0, and 44.0 for Africans, Europeans, and Native Americans, respectively. Because the mean locus specific branch length for European ancestry was lower than for African or Native American ancestry, there are 202 European AIMs with a median LSBL *F_st_* of 0.37 (25: 75 percentiles 0.35–0.41) and a median LSBL *I_n_* of 0.21 (25:75 percentiles 0.20–0.23). There are 115 African AIMs with a median LSBL *F_st_* of 0.63 (25: 75 percentiles 0.61–0.66) and a median LSBL *I_n_* of 0.37 (25:75 percentiles 0.36–0.40). The 129 Native American AIMs have a median LSBL *F_st_* of 0.56 (25: 75 percentiles 0.54–0.61) and a median LSBL *I_n_* of 0.33 (25:75 percentiles 0.32–0.36). The informativeness of the AIMs panel is summarized in Table 1. The lower informativeness of European-specific AIMs is likely because European populations are geographically and genetically intermediate to African and Native American populations [18], [19]. Consequently, more European AIMs were needed to provide balanced discriminatory power.

**Table 1:** Characteristics of the AIMs panel.

Population	Number of AIMs	Cumulative LSBL F_st_	Cumulative LSBL I_n_	LSBL F_st_	LSBL I_N_
				(mean ± sd; median, 25:75)	(mean ± sd; median, 25:75)
African	115	73.0	43.8	0.64±0.05;0.63, 0.61: 0.66	0.38±0.03;0.37, 0.36: 0.40
European	202	77.9	44.0	0.39±0.05;0.37, 0.35: 0.41	0.22±0.03;0.21, 0.20: 0.23
Native American	129	74.5	44.0	0.58±0.05;0.56, 0.54: 0.61	0.34±0.03;0.33, 0.32: 0.36

### Validation of the panel of AIMs

**Figure 1:**
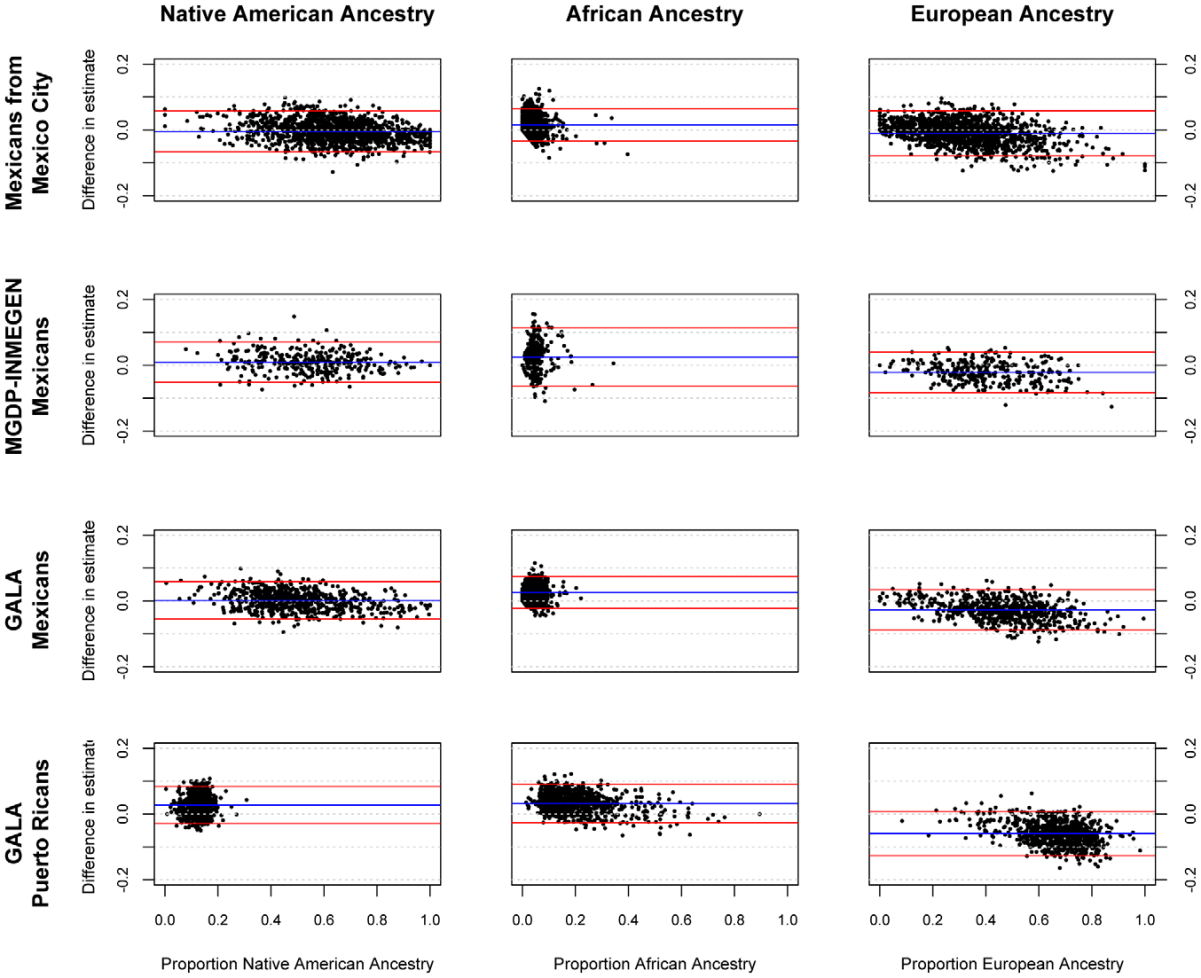
Bland-Altman plots showing error in individual ancestral estimates using AIMs to ancestral estimates using GWAS data. The x-axis shows the ancestry estimate using GWAS data; the y-axis shows the difference in estimates between GWAS and AIMs data using the 425 AIMs genotyped in the GALA Mexicans and Puerto Rican samples, 314 AIMs for the Mexico City sample, and 398 AIMs for the MGDP-INMEGEN sample.

Figure 1 shows the accuracy of the ancestry estimates obtained with the AIMs panel for four Latin American samples. The individual ancestry estimates based on the AIMs panel were compared to the estimates based on genome-wide data. Generally, there was strong concordance between ancestry estimates using the AIMs and using GWAS data. There is a slight systematic underestimate of European ancestry in all four populations tested, and a slight overestimate of African ancestry. Table 2 summarizes the performance of ancestry estimates for in the four admixed samples. The correlation (R^2^) between ancestry estimates using AIMs and ancestry from GWAS data is high in most cases, especially for Native American and European ancestry in all three Mexican samples and European and African ancestry in the Puerto Rican sample. The correlation coefficient was lower for estimates of ancestry where there was less variance in the true ancestral proportion.

**Table 2:** Validation of the AIMs panel compared to ancestry estimates using GWAS data.

Sample Ancestry	Mean ancestry estimate (with GWAS)	Correlation R^2^	Mean error ± sd	Mean discordance	Root mean square error
**Mexico City**					
Native American	0.642	0.968	−0.005 (±0.032)	0.025	0.032
European	0.324	0.956	−0.010 (±0.034)	0.028	0.036
African	0.035	0.555	0.015 (±0.025)	0.023	0.029
**MGDP-INMEGEN**					
Native American	0.544	0.966	0.009 (±0.031)	0.025	0.032
European	0.402	0.964	−0.022 (±0.031)	0.031	0.038
African	0.054	0.722	0.012 (±0.023)	0.020	0.026
**Mexico GALA**					
Native American	0.496	0.972	0.002 (±0.029)	0.023	0.029
European	0.458	0.967	−0.027 (±0.031)	0.033	0.041
African	0.046	0.558	0.026 (±0.025)	0.029	0.035
**Puerto Rico GALA**					
Native American	0.124	0.603	0.027 (±0.029)	0.033	0.040
European	0.670	0.914	−0.059 (±0.034)	0.060	0.068
African	0.206	0.942	0.032 (±0.030)	0.036	0.044

### Use of AIMs panel subsets to control for population stratification

**Figure 2:**
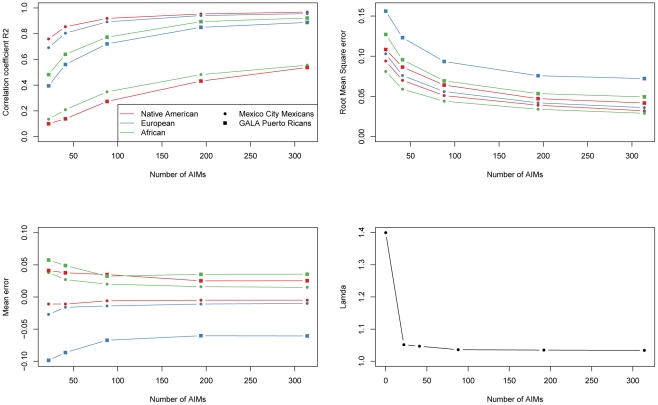
Performance of nested subsets of AIMs.

We investigated the effect of the number of AIMs on the accuracy of the estimates of ancestry, using the parents of Puerto Rican subjects with asthma from the GALA study (n = 803) [20] and the sample from Mexico City, which includes 967 cases and 343 controls from a Type II Diabetes study [21], [22]. We compared the estimates of ancestry based on genome-wide data with the estimates obtained with different subsets of AIMs (314, 194, 88, 41 and 22). For this analysis, we first started with the 314 AIMs that were genotyped in this sample. We produced nested subsets of AIMs by progressively reducing the number of AIMs, keeping only the most informative markers, and ensuring that the final panel of AIMs was balanced (e.g. each panel has approximately the same ancestry information content for each ancestral group). Ancestry estimates were estimated with the program ADMIXTURE with ancestral genotype data. Table 3 and Figure 2 depict the correlation (R^2^) between the genome-wide estimates and the estimates based on the panel of AIMs, as well as the mean differences, mean absolute differences and root mean square errors. As expected, reducing the number of AIMs in the panel results in decreasing correlation and increasing error of the ancestry estimates compared to the estimates produced with genome-wide data. Performance of the 194 AIMs panel, and to a lesser extent the 88 AIMs panel is comparable to performance of the 314 AIMs panel. The correlations between the estimates based on 22 AIMs and those based on genome-wide estimates are considerably worse, particularly for the estimates of African ancestry in Mexicans and Native American ancestry in Puerto Ricans, which are the ancestral components with the least amount of variance between subjects.

**Table 3:** Performance of nested subsets of AIMs.

Sample	Correlation R^2^	Mean error	Mean discordance	RMSE
**314 AIMs in Mexico City Mexicans**				
Native American	0.97	−0.005	0.025	0.032
European	0.96	−0.010	0.028	0.036
African	0.56	0.015	0.023	0.029
**314 AIMs in GALA Puerto Ricans**				
Native American	0.54	0.025	0.034	0.042
European	0.89	−0.061	0.063	0.072
African	0.92	0.035	0.041	0.049
**194 AIMs in Mexico City Mexicans**				
Native American	0.95	−0.005	0.031	0.039
European	0.94	−0.011	0.033	0.042
African	0.48	0.016	0.026	0.034
**194 AIMs in GALA Puerto Ricans**				
Native American	0.43	0.025	0.034	0.042
European	0.85	−0.060	0.063	0.072
African	0.89	0.035	0.044	0.053
**88 AIMs in Mexico City Mexicans**				
Native American	0.92	−0.006	0.040	0.051
European	0.89	−0.014	0.044	0.056
African	0.35	0.020	0.034	0.044
**88 AIMs in GALA Puerto Ricans**				
Native American	0.27	0.035	0.052	0.064
European	0.72	−0.067	0.077	0.093
African	0.77	0.032	0.055	0.069
**41 AIMs in Mexico City Mexicans**				
Native American	0.85	−0.011	0.056	0.070
European	0.80	−0.016	0.061	0.076
African	0.21	0.027	0.044	0.059
**41 AIMs in GALA Puerto Ricans**				
Native American	0.14	0.038	0.069	0.086
European	0.56	−0.086	0.101	0.123
African	0.64	0.049	0.076	0.096
**22 AIMs in Mexico City Mexicans**				
Native American	0.76	−0.011	0.075	0.094
European	0.69	−0.027	0.081	0.103
African	0.14	0.038	0.059	0.081
**22 AIMs in GALA Puerto Ricans**				
Native American	0.10	0.041	0.086	0.108
European	0.39	−0.099	0.125	0.156
African	0.48	0.057	0.101	0.127

We evaluated the utility of the different panels of AIMs to control for the effects of population stratification in the Mexico City sample, which had previously been shown to have significant population stratification [22]. The average Native American ancestry in the cases was estimated to be 66% versus 57% in the control group. We carried out a logistic regression analysis to test the association of approximately 315,000 common markers with type 2 diabetes, including as covariates sex and age, or alternatively, sex, age and the ancestry estimates obtained with 314, 194, 88, 41 and 22 AIMs. We then prepared quantile-quantile (QQ) plots comparing the p values obtained in the logistic association tests with the values expected under the null model of no association (See Figure S1). The extent of population stratification was quantified by the inflation factor lambda [23], using the program WGAViewer. Under the model conditioning by sex and age, there was a strong departure of the observed and expected p-values. The value of lambda was 1.4, indicating grossly inflated false-positive rates. As seen in Figure 2, adding ancestry estimates to the model dramatically reduced the inflation factor: reducing lambda to 1.04 using genome-wide estimates of ancestry. Using AIMs panels of 314 AIMs, 194 AIMs and 88 AIMs produced nearly equal reductions in lambda. Performance using smaller AIMs panels still resulted in a marked decrease in the inflation factor: for the 41 and 21 AIMs panels lambda was 1.05.

### Ancestry estimates for 18 populations in the Americas

The panel of AIMs was carried forward to genotype a total of 373 individuals from 18 populations throughout the Americas using the Sequenom platform. Generally speaking, the platform performed well, though 75 SNPs were excluded due to lower call rates (all samples included). The final analysis was based on 325 markers. Among the SNPs meeting quality control criteria, the average call rate was 91.7% (max value 99.5% and min value 55.1%, all samples included). Two additional populations (Coyas and Mapuches) were genotyped but excluded from the analysis due to the low quality of the samples. Four additional individuals were excluded due to genotyping call rates of <90%.

**Figure 3:**
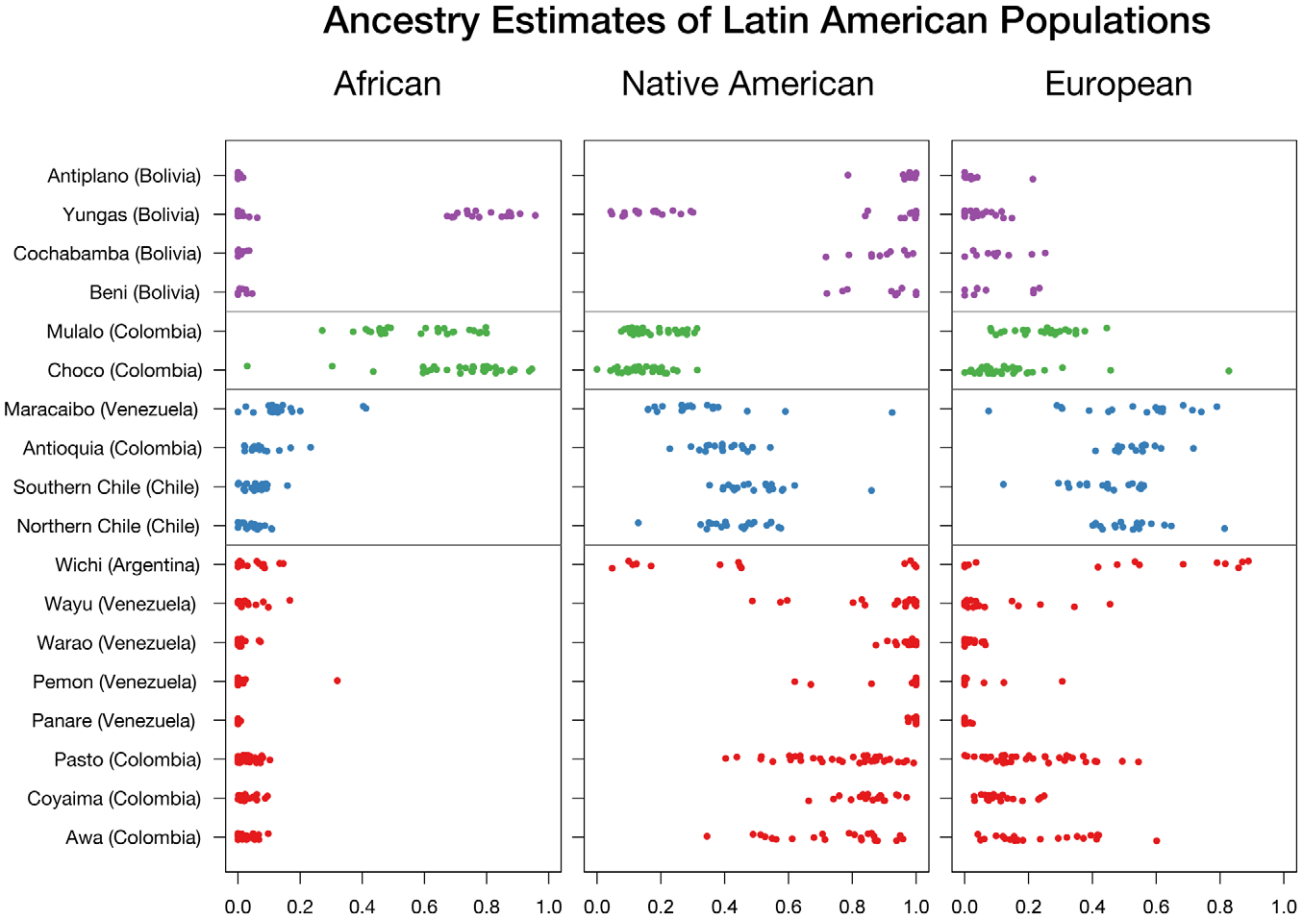
Ancestry estimates of Latin American populations.

Table 4 summarizes the ancestral estimates obtained for the 18 populations characterized and Figure 3 shows one-dimensional scatter plots of ancestry for each ancestral component in each sample. As expected, most of the indigenous populations have high Native American ancestry, with a median (25:75 percentile) Native American ancestry of 0.80 (0.57: 0.87) for Colombian Awa, 0.86 (0.83: 0.89) for Colombian Coyaima, 0.83 (0.64: 0.87) for Colombian Pastos, 1.0 (1.0: 1.0) for Venezuelan Panare and Pemon, 0.99 (0.97: 1.0) for Venezuelan Warao, and 0.97 (0.84: 1.0) for Venezuelan Wayu. The Wichi from Argentina had relatively lower Native American ancestry, which was estimated as 0.41 (0.12: 0.84). The Bolivian individuals recruited in the Beni and Cochabamba Departments, as well as those from the Altiplano region of the La Paz Department also showed high Native American ancestry proportions. The median Native American ancestries for these samples were 0.94 (0.78: 0.96), 0.90 (0.86: 0.95) and 0.98 (0.96: 1.0), respectively. In contrast, in the Bolivian sample from the subtropical Yungas region, which is known for the presence of scattered Afro-Bolivian communities, many individuals had relatively high African ancestry (>0.6), whereas other individuals showed primarily Native American ancestry (>0.8) (Table 4 and Figure 3). The median African and Native American ancestries observed in the Yungas sample were 0.70 (0.01: 0.82) and 0.25 (0.13: 0.97), respectively.

**Table 4:** Ancestry of Latin American populations.

Population	Country	Sample size	Native American Ancestry	European Ancestry	African Ancestry
**Awa**	Colombia (Southern)	22	0.80, 0.57: 0.87	0.17, 0.12: 0.37	0.02, 0.0: 0.05
**Coyaima**	Colombia (Central)	19	0.86, 0.83: 0.89	0.09, 0.07: 0.13	0.02, 0.01: 0.05
**Pastos**	Colombia (Southern)	36	0.83, 0.64: 0.87	0.16, 0.12: 0.31	0.02, 0.0: 0.04
**Panare**	Venezuela (Amazon)	20	1.00, 1.00: 1.00	0.0, 0.0: 0.0	0.0, 0.0: 0.0
**Pemon**	Venezuela (Amazon)	20	1.00, 1.00: 1.00	0.0, 0.0: 0.0	0.0, 0.0: 0.0
**Warao**	Venezuela (Amazon)	20	0.99, 0.97: 1.00	0.01, 0.0: 0.02	0.0, 0.0: 0.01
**Wayu**	Venezuela (North)	20	0.97, 0.84: 0.99	0.02, 0.0: 0.08	0.02, 0.0: 0.03
**Wichi**	Argentina	14	0.41, 0.12: 0.84	0.54, 0.13: 0.81	0.05, 0.01: 0.08
**Maracaibo**	Venezuela	20	0.28, 0.25: 0.36	0.60, 0.44: 0.62	0.12, 0.11: 0.15
**Northern Chile**	Chile	20	0.46, 0.37: 0.50	0.51, 0.43: 0.55	0.05, 0.03: 0.07
**Southern Chile**	Chile	20	0.51, 0.43: 0.55	0.45, 0.38: 0.53	0.06, 0.03: 0.08
**Antioquia**	Colombia	19	0.39, 0.35: 0.46	0.52, 0.48: 0.56	0.06, 0.04: 0.08
**Antiplano**	Bolivia	11	0.99, 0.98: 1.00	0.0, 0.0: 0.02	0.0, 0.0: 0.01
**Chocó**	Colombia	35	0.13, 0.10: 0.18	0.10, 0.07: 0.16	0.76, 0.64: 0.83
**Mulaló**	Colombia	28	0.18, 0.12: 0.26	0.25, 0.19: 0.20	0.54, 0.46: 0.69
**Beni**	Bolivia	10	0.94, 0.78: 0.96	0.04, 0.03: 0.22	0.01, 0.0: 0.03
**Cochabamba**	Bolivia	12	090, 0.86: 0.95	0.09, 0.05: 0.13	0.0, 0.0: 0.01
**Yungas**	Bolivia	27	0.25, 0.13: 0.97	0.03, 0.0: 0.05	0.70, 0.01: 0.82

Ancestries are given in median and 25^th^:75^th^ percentiles.

The two Afro-Colombian samples included in this study had a median African ancestry of 0.76 (0.64: 0.83) for Chocó and 0.54 (0.46: 0.69) for Mulaló. Finally, the Mestizo samples from Colombia, Venezuela and Northern and Southern Chile showed a relatively high dispersion in Native American and European admixture proportions. With the exception of some Venezuelans from Maracaibo, on the Caribbean coast, most of these individuals had small (<10%) African contributions.

**Figure 4:**
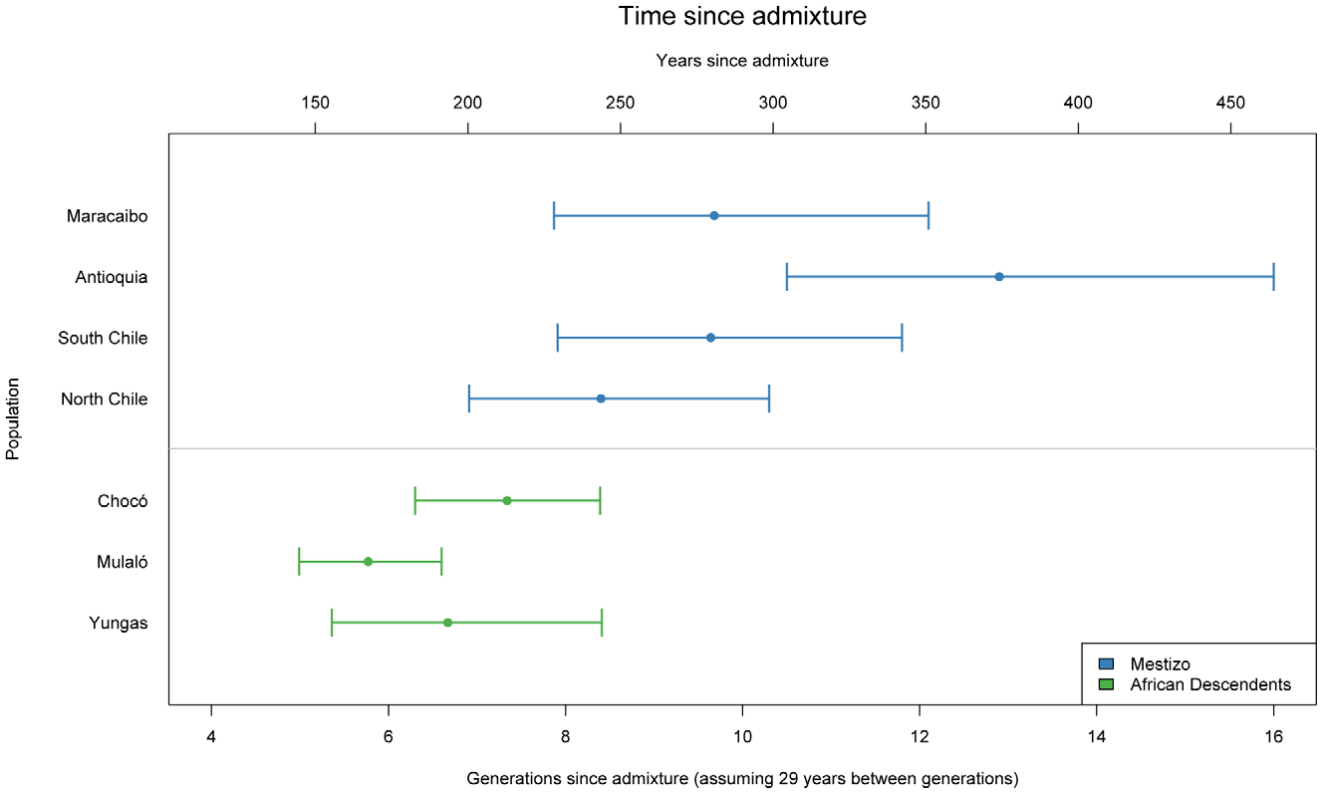
Time since admixture for Mestizo and African descendent populations.

We also estimated the average number of generations since admixture for the Mestizo and African descendant samples (Figure 4). Generally speaking, in the Afro-Colombian and Afro-Bolivian samples, the estimated time since admixture was 6.7 generations (95% credible interval: 5.4–8.4) for the Yungas, 5.8 generations (95% credible interval: 5.0–6.6) for the Mulaló and 7.34 generations (95% credible interval: 6.3–8.4) for the Chocó. In contrast, the estimates of time since admixture for the Mestizo samples were higher; the estimated time since admixture was 8.4 generations (95% credible interval: 6.9–10.3) for the Northern Chileans, 9.6 generations (95% credible interval: 7.9–11.8) for the Southern Chileans, 12.9 generations (95% credible interval: 10.5–16.0) for the Colombians, and 9.7 generations (95% credible interval: 7.9–12.1) for the Venezuelans.

## Discussion

In this study, we developed, validated, and tested a novel panel of AIMs designed to accurately estimate the ancestral components (African, European, and Native American) of contemporary Latin American populations. We developed a new algorithm (provided in the web resources online) capable of taking genome-wide data from multiple populations within each continental group and identifying the most informative, well-balanced and portable markers to estimate ancestry proportions.

The ancestral samples used to identify the AIMs represented a wide variety of populations within each continental group. Specifically, we used six samples from Mesoamerica and the South American Andes as representatives of the ancestral Native American populations that make up modern Latin Americans. Our Native American samples had a median Native American ancestry of 97.7% (25: 75 range 93.2% to 100%) based on ancestry ascertainments using genomewide data. Given the history of European colonization in the Americas, a small amount of European genetic admixture (2.3%, 1×10^−5^: 6.2%) is not surprising. However, a small amount of European admixture would be expected to result in an underestimate of the information content of our AIMs. Although we did not include Native American populations from English-speaking North America for our analysis, our selection of markers excluded those with significant heterogeneity between Native American populations. Thus, we have no reason to believe the markers cannot be applied to North American populations, though the use of these markers for populations outside of Latin America should be pursued with caution.

We also included two samples from Africa (Yoruba from Nigeria and Luhya from Kenya, in East Africa). Historical records and genetic analyses indicate that most of the slaves imported into the Americas originated in West Africa [24]. Although it would have been ideal to include multiple West African ancestral populations, we included the Luhya sample in our study because unlike the Yoruba, who are descendants of the Benue-Congo subfamily of the Niger-Congo language family, the Luhya are a Bantu-speaking population, and many of the enslaved Africans brought to the Americas were Bantu speakers. Multiple studies show that the Luhya and other Bantu-speaking groups from East Africa are more closely related to West African Bantu speakers than to other East African ethnic groups [24], [25]. In addition, a small but significant number of slaves originated in Southeastern Africa [26], [27], [28]. Finally, we used three European samples to estimate ancestral frequencies in Europe. Importantly, samples from Italy and the Iberian Peninsula, which have been the largest sources of European migrants to Latin America, were included in this analysis.

By excluding markers with significant within-continent heterogeneity, the selected panel of AIMs should be broadly portable to populations from throughout the Americas. Moreover, the exclusion of markers exhibiting substantial within-continent heterogeneity serves to ensure that there is relatively little bias in the estimates of ancestral allele frequency. This is because any bias would have had to occur in all of the ancestral populations within a given continent, at a similar magnitude and in the same direction.On the other hand, by design, the AIMs panel would not be expected to differentiate within-continent population substructure. Indeed, we found that the eight Native American populations genotyped with the AIMs panel were indistinguishable in principal component space beyond the first principal component, which represented the degree of European admixture (data not shown). There are several reasons we chose to exclude markers that could have potentially been used to differentiate within-continent substructure. First, the principal reason for designing this panel was for identifying continental ancestry proportions in admixed samples, as continental admixture is the most important source of population structure in Latin Americans. Secondly, because we had a limited number of Native American ancestral groups available for study, we would have only been able to generate AIMs that distinguished Mesoamerican populations from Andean populations. Third, the use of heterogeneity filters was an important element of quality control, as it served to filter out alleles with extreme frequencies due to bias. Finally, because the genetic differences within continental groups are smaller than between continental groups, we would have required many more markers to accurately determine within-continent substructure.

We validated the panel of AIMs by comparing ancestry estimates derived from the subset of AIMs to estimates derived from genome-wide data in four Latin American populations, three from Mexico and one from Puerto Rico. Overall, the ancestral estimates for both Puerto Ricans and all Mexican groups were consistent with previously published literature [29]. Specifically, Bryc et al found that Puerto Ricans had 23.6%±12% African ancestry, consistent with our finding of 20.6%±12.3% and Mexicans had 5.6%±2% African ancestry, consistent with our findings of between 3.5%±3.1% and 5.4%±3.6%. The Native American component in the three Mexican populations (64.2%±17.6%, 54.4%±16.9%, and 49.6%±17.4% in Mexicans from Mexico City, INMEGEN, and GALA studies, respectively) is also consistent with results obtained by Bryc et al (50.1%±13%) and in a study by Silva-Zolezzi *et al* of diverse Mexican Mestizo populations (55.2%±15.4%) [30].

There was strong correlation between ancestral estimates obtained from the AIMs panel and those obtained from GWAS, providing strong support for the use of the AIMs panel to accurately estimate ancestry. For over 95 percent of the samples, the estimates of ancestry using AIMs were within 10% of the value obtained using GWAS data.

The correlation was lower for the minor ancestral components (African ancestry in Mexican populations and Native American ancestry in Puerto Rican populations). This reflected the more limited between-subject variance in the minor ancestral component. Since the coefficient of determination (R^2^) represents the proportion of variance in the outcome variable (in this case, the true measure of ancestry), explained by the predictor (estimates of ancestry using AIMs), in cases where there is more limited variance in the outcome variable such as estimates of African ancestry in Mexicans and Native American ancestry in Puerto Ricans, we observe a lower R^2^. Nonetheless, measures of individual error in estimate, such as the root mean squared error, are comparable for all three ancestral estimates in both Puerto Ricans and Mexicans, suggesting that the panel performs consistently across all ancestral components, and in most cases, the estimate of ancestry using AIMs lies within 10% of the true measure of ancestry, as can be seen in Figure 1.

The small systematic errors in the estimation of ancestry with AIMs are likely due to the bounding of ancestry proportions at 0 and 1. The most a minor ancestral component can be underestimated is equal to its true value (for example, an ancestral estimate of 4% can at most be underestimated by 4%, if it is estimated to be 0%), but it can be overestimated much more substantially. Conversely, the major ancestral component cannot be overestimated by more than the difference between 100% and its true value, but it can be significantly underestimated. This effect is most notable in Figure 1 with African ancestry in Mexicans from Mexico City, where the bounding is visible as what appears to be a line with a slope of −1 that forms the lower limit of error estimates for ancestry proportions less than 0.05. The slight increase in noise from AIMs panels compared to genome-wide estimates should then result in overestimates of minor components and underestimates of major components, consistent with observation.

We used the panel of AIMs to genotype 373 individuals from 18 Latin American populations. The samples were very diverse, and included individuals from several indigenous groups, African descendants and Mestizos from five different countries. Generally speaking there is strong concordance between ethnicity and admixture estimates. Specifically, seven out of eight indigenous samples showed a high degree of Native American ancestry. In particular, the four isolated groups from Venezuela (Warao, Panare and Pemon from the Amazon and Wayu from the northwestern region of Venezuela) showed very little evidence of European or African admixture. The three indigenous groups from Colombia (Coyaima, Pastos and Awa) had average Native American proportions higher than 80%, and a relatively small European contribution. That our AIMs panel could effectively estimate ancestry in lowland South American Native American populations (such as those in Venezuela) despite the fact that our AIMs were derived from Mesoamerican and Andean populations is reassuring and demonstrates that our strategy of excluding markers with significant heterogeneity ensures the generalizability of the markers. The indigenous Wichi from Argentina had considerably lower Native American ancestry and higher European ancestry (0.41 and 0.54, respectively) than the indigenous groups from Venezuela and Colombia. This is consistent with a recent study of Y-chromosomes that found widespread European paternal ancestry among Amerindian groups, including the Wichi, in Argentina [31]. Interestingly, we observed cryptic and previously unreported European admixture in the two isolated Indigenous populations from Southern Colombia, a fairly common phenomenon in Native American populations [32].

In Bolivia, we found that the individuals from the Departments of Beni, Cochabamba and the Altiplano region of the La Paz Department had, on average, high Native American contributions. However, in the subtropical area of Yungas, many of the individuals recruited in the small community of Tocaña and one of the individuals recruited in the nearby town of Coroico had high African ancestry (median = 0.78, 0.74: 0.80). The subtropical Yungas region is home to several scattered Afro-Bolivian communities. These Afro-Bolivians are the descendants of African slaves who were brought to work on the Potosi mines and coca plantations [33]. Our data indicate that the admixture process in this Afro-Bolivian community has been primarily with the indigenous groups living in this region (median Native American ancestry = 0.13, 0.09: 0.20, median European ancestry = 0.04, 0.02: 0.06).

Two additional groups of African descent were included in this study, the Mulaló and Chocó from Colombia. African slaves were brought to Colombia early during the colonial period for gold mining, sugar cultivation, and cattle ranching. The proportion of African ancestry in these two Afro-Colombian groups was slightly lower than in the Afro-Bolivian community (0.54, 0.46: 0.69 in the Mulaló and 0.76, 0.64: 0.83 in the Chocó). Unlike the Afro-Bolivian sample from the Yungas region, in which most of the non-African contribution came primarily from indigenous groups, the two Afro-Colombian samples had similar European and Native American ancestral contributions (Table 4). This highlights the diverse history of admixture in different areas within Latin America. Similar observations have been reported by Castro de Guerra and colleagues [34], [35], which compared two African derived populations in Venezuela and found that one, the Patenemos, showed mostly European ancestry, while the other population, Ganga, was principally admixed with Native American ancestry. We estimated that the time since admixture in the three samples of African descent is approximately 6 to 7 generations, corresponding to between 174 and 203 years, indicating that, the admixture process in these groups has been relatively recent. Though the point estimates of the years since admixture are approximately 50 to 100 years after the time when slaves were introduced into the region for gold and silver mining, because of the wide credible intervals, our estimates are not inconsistent with the historical record [36].

Our samples from the four Mestizo populations from Chile, Colombia, and Venezuela showed a wide variability in the ancestral proportions, though the primary ancestral contributions were European and Native American. Only some of the subjects from Maracaibo, on the Caribbean coast of Venezuela, had greater than 10% African ancestry, as did some of the Puerto Rican subjects used to validate the AIMs. This is unsurprising, given that the rest of our Mestizo populations are from Mexico, Chile and the Northwest of Colombia, areas where the slave trade was not prominent. This is consistent with the findings of Wang *et al*, who examined thirteen Mestizo populations in Latin America and found extensive variation in Native American and European ancestry and relatively low levels of African ancestry [37]. We estimated between eight and thirteen generations since admixture for the mestizo samples, corresponding to between 230 and 375 years, reflecting the earlier settlement of substantial contingents of Europeans in Colombia than in Chile [38].

One striking finding in this paper is the rich ancestral variation in the Americas, even within a single country. For example, among the six Colombian populations examined (three Native American populations, one Mestizo population, and two Afro-Colombian populations), median Native American ancestry varied between 0.13 in the Chocó and 0.86 in the Coyaima, African ancestry varied between 0.02 in the three Amerindian populations and 0.74 in the Chocó, and European ancestry varied between .09 in Coyaima and 0.52 in the Colombian mestizos. Likewise, even among the Bolivians in a single administrative department (state), there was a wide variation in African and Native American ancestry (Figure 3). These patterns of variation in ancestry within small regions seem to be a common feature across the Americas and have also been recently found in the island of Puerto Rico [3]. This has broad implications for genetic association studies in Latin American subjects, as there is a strong potential for population stratification, even in samples from a single country or a single administrative region within a country, and emphasizes the importance of incorporating ancestry estimates into future genetic association studies in these populations. We anticipate the primary use of this panel of AIMs will be to control for population stratification in genetic association and medical genetic studies. Thus, the ability of our panel of AIMs to effectively control for population stratification, as evidenced by its ability to reduce the genomic inflation factor in a highly stratified study of Type II diabetes in Mexican subjects, is an important source of validation. Even small subsets of AIMs from the panel adequately control for population stratification, suggesting that the panel should adequately cope with the significant patterns of variation in ancestry seen in Latin American. Nonetheless, because the panel of markers is not designed to identify within-continent heterogeneity, it is possible that it may not adequately control for finer population substructure.

In summary, we have developed and validated a panel of 446 AIMs to estimate European, Native American and African admixture proportions. The markers were selected to have low heterogeneity within continents, in order to be portable throughout the Americas. This panel was specifically designed to provide accurate individual admixture estimates and to control for the effects of population stratification in association studies in admixed populations. The use of this panel will minimize the risk of false positives in candidate gene studies, or in research efforts designed to replicate signals identified in genome-wide association studies, even in studies with substantial population stratification.

Our analysis of subsets of this panel has shown that to successfully control for population stratification in association studies, panels with 314, 194 and even 88 AIMs provide adequate estimates of the ancestral proportions with greatest variance that are strongly correlated with the genome-wide estimates (R^2^ of 0.9 or higher) and have mean absolute error under 5%. Panels with 314, 194 and 88 AIMs all adequately controlled for the effects of population stratification in the Mexico City sample. The inflation factor (lambda) was reduced from 1.40 when using sex and age as covariates, to less than 1.04 when incorporating ancestry estimates based on genome-wide data and panels of 314, 194 and 88 AIMs, and reasonable control for population stratification could be achieved with even smaller panels.

There are several important limitations to our AIMs panel. It is important to point out that the density of the markers in this panel is inadequate for admixture mapping, although the enclosed Python script could be used to identify a sufficient number of AIMs to perform an admixture mapping study [39]. Several research groups have already made available denser genome-wide panels of AIMs for admixture mapping in African Americans [40], [41], [42] and Hispanics [43], [44], [45], although none of these panels was designed for admixture models including three ancestral populations. The AIMs were selected for their information content on African, European and Native American ancestry. These have been the major population groups contributing ancestry in the Americas since the 15^th^ century. However, in many locations within the Americas, the history of human migration and admixture has been extremely complex, and has involved other population groups, such as East Asians and South Asians [46]. This panel of AIMs should be applied cautiously to populations (or individuals) with such complex admixture histories. Finally, while the panel has been validated to study the history of recent admixture in Latin America, it is unlikely to be effective in inferring finer scale population history.

As with all panels of AIMs, our panel is vulnerable to ascertainment bias, because the AIMs were selected to maximize the difference in continental ancestral allele frequencies. However, there are several factors that minimized the impact of this bias. First, we had a large sample size of all ancestral groups, particularly the European populations. Since the standard error of the estimate of allele frequency is inversely proportional to the square root of the number of individuals, the large sample sizes minimize the standard error in allele frequency estimates. Secondly, we used multiple populations within each continental group, and excluded any markers that showed large amounts of heterogeneity among ancestral groups within each continent. Thus, samples biased in one population (due to chance or genotyping error) are likely to have been filtered out. Finally, when we applied our panel to new populations, it produced credible ancestry estimates, which compare favorably to ancestry ascertained from genomewide data not subject to ascertainment bias.

This panel is intended to be an important resource for the community and we have provided both the source code for the algorithm to generate the AIMs, as well as allele frequency data and anonymized ancestral African, European, and shuffled Native American genotype information. We hope that investigators can use the selected panel of AIMs, which can be easily genotyped on readily available platforms, as a cost-effective tool to estimate continental ancestry in modern populations of the Americas.

## Materials and Methods

### Ethics statement

Informed consent was obtained for all subjects in all phases of this study, with input from local communities. These studies were approved by local institutional review boards and the relevant offices at each institution contributing samples (detailed information on approvals and consents for all samples available in Text S1).

### Ancestral samples and genotyping

Subjects representing the three main continental ancestral groups making up modern Latin American populations were obtained from a variety of sources. Hapmap Phase III genotype data for African and European populations was downloaded for this project, including West African (Yoruba in Ibadan, Nigeria, YRI) and East African (Luhya in Webuye, Kenya, LWK) as well as Northern European (Utah residents with ancestry from Northern and Western Europe, CEU) and Southern European (Toscani in Italy, TSI) individuals [47], [48]. For populations including parent/child trios or duos (CEU, YRI), only genotypes from the parents were used. In addition, known cryptically related individuals were removed [49]. Genotyping data for Europeans was further supplemented by a cohort of 619 samples of Spanish individuals, genotyped on the Affymetrix SNP 6.0 platform.

One hundred and thirty-one Native American subjects, from Mesoamerica (Nahua from Central Mexico, n = 14, Zapotecas from Oaxaca, Mexico, n = 21, and Maya from Campeche, n = 25) [16], [30], [50], from the Sierra Madre Occidental region (Tepehuanos from Durango in Northern Mexico, n = 22) and South America (Aymara from La Paz, Bolivia, n = 25, and Quechua from Cerro de Pasco, Peru, n = 24) [14], [51] were used to determine Native American allele frequencies. These populations were genotyped either on the Affymetrix SNP 6.0 or on two platforms, the Affymetrix SNP 500K and the Illumina 550.

A summary of the populations used for this study and genotyping platforms is given in Table 5. Although, additional Native American subjects with genomewide data are available from the Human Genome Diversity Panel, these subjects were genotyped on the Illumina HumanHap 650k, and the intersection with the genotyping platforms used in our samples would have left fewer markers to be evaluated.

**Table 5:** Ancestral populations used for this study.

Population	Designation	Sample size	Platform(s)
**Utah residents with ancestry from Northern and Western Europe (HapMap Phase III)**	CEU	56	Affymetrix 6.0/Illumina 1M
**Toscani in Italy (HapMap Phase III)**	TSI	44	Affymetrix 6.0/Illumina 1M
**Spaniards from Spain**	SPAIN	619	Affymetrix 6.0
**Yoruba in Ibadan, Nigeria (HapMap Phase III)**	YRI	53	Affymetrix 6.0/Illumina 1M
**Luhya in Webuye, Kenya (HapMap Phase III)**	LWK	50	Affymetrix 6.0/Illumina 1M
**Aymara from La Paz, Bolivia**	AYMARA	25	Affymetrix 6.0
**Quechua from cerro de Pasco, Peru**	QUECHUA	24	Affymetrix 6.0
**Nahua from Central Mexico**	NAHUA	14	Affymetrix 6.0
**Maya from Campeche, Mexico**	MAYAS	25	Affymetrix 500K/Illumina 550K
**Tepehuano from Durango, Mexico**	TEPHUANOS	22	Affymetrix 500K/Illumina 550K
**Zapoteca from Oaxaca, Mexico**	ZAPOTECAS	21	Affymetrix 500K/Illumina 550K

### Quality control

Four major quality control tests were performed on the data using the program plink [52]. Individuals were excluded if they had greater than 10% missing alleles, if they were known to be related, or showed cryptic relatedness. For Native American populations, pairwise individuals were considered to have cryptic relatedness if their IBS scores showed a Z1>0.15 or a Z2>0.03 or if they had a proportion IBD (pi hat)>0.08 [53]. Europeans and African individuals were considered cryptically related if they had a Z1>0.03 or Z2>0.03, or if they had a proportion IBD>0.03. SNPs were included if the genotyping rate was greater than 90% and excluded if they failed a χ^2^ test for Hardy-Weinberg equilibrium at a significance threshold of 10^−5^.

### Stage one: AIM selection

**Figure 5:**
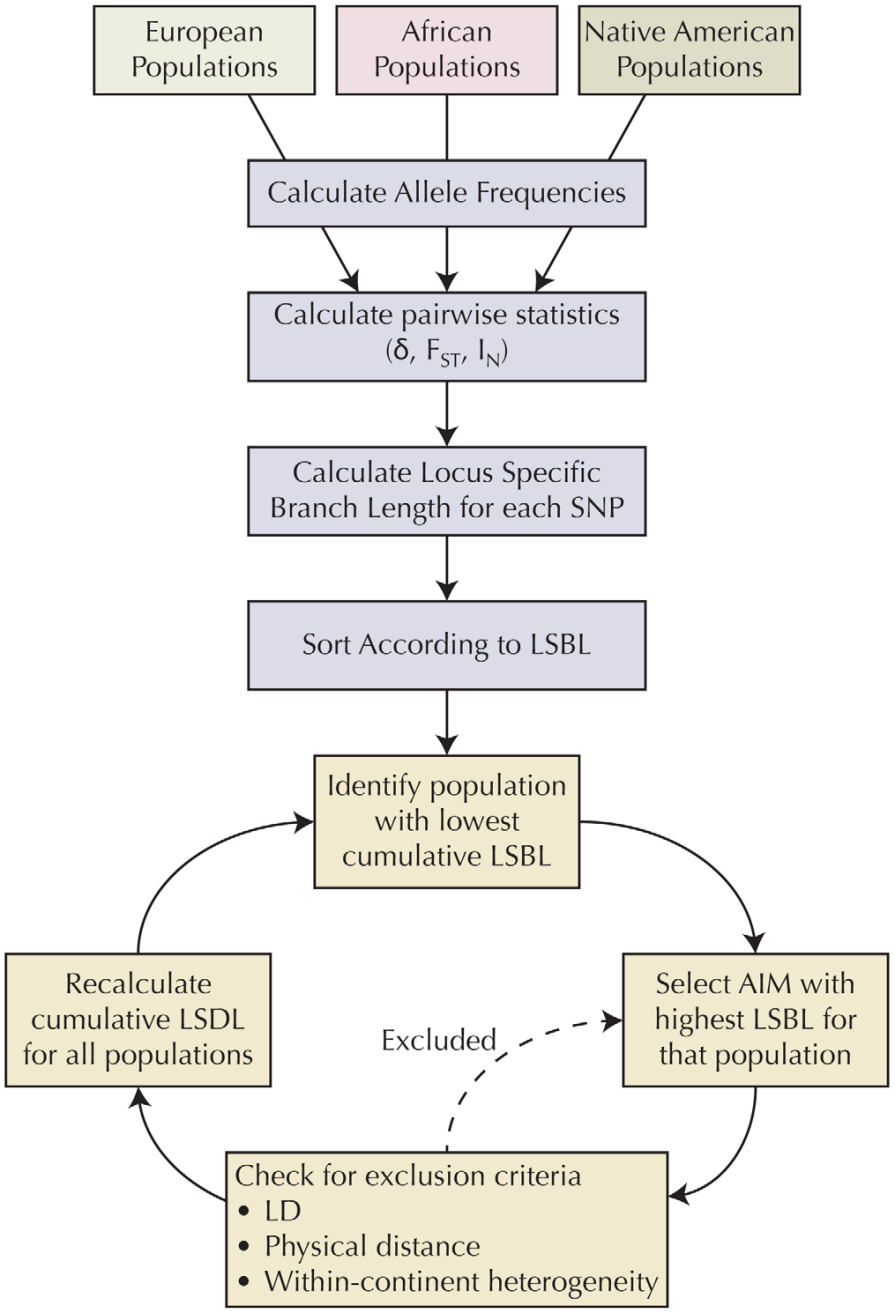
Algorithm for selecting AIMs.

Markers representing the intersection of the genotyping platforms used to genotype the ancestral populations, which met quality control criteria (n = 319,665) were used as a basis for selecting AIMs. Figure 5 summarizes the methodology used to select AIMs. For each SNP for each ancestral group, allele frequency was calculated with the program plink [52]. For each marker, statistics of informativeness, including delta, *F_st_* [54], and Rosenberg's informativeness for assignment statistic *I_n_* [55] were calculated between each pair of ancestral populations (African/European, European/Native American, and African/Native American) based on reference allele frequencies. Locus specific branch length (LSBL) [56] statistics were created for each population and each statistic of informativeness to translate the pairwise metrics into a population-specific statistic. A balanced set of AIMs was selected by ensuring that the cumulative LSBL for each population was approximately equal. At each stage, we selected the polymorphism with the highest LSBL for the population with the lowest cumulative LSBL that met the inclusion criteria. Polymorphisms were excluded if they were in linkage disequilibrium (r^2^≥0.1) or within a predefined physical distance (≤500 kb pairs) of previously selected AIMs. This ensures maximum independent informativeness and that the AIMs were well distributed throughout the genome. In addition, in order for potential AIMs to be applicable to all subpopulations within a continental group, potential AIMs were also excluded if there was evidence of significant allele frequency heterogeneity between the samples representing each ancestral group (χ^2^ p-value<0.01). A script in the Python programming language that implements this algorithm and ancestral population allele frequency data are available for download.

### Stage two: Validation of the panel of AIMs

In order to validate the panel of AIMs, estimates of ancestry using the panel were compared to estimates of ancestry using genome-wide data. Four admixed samples were used for validation. The first two datasets were parents of Puerto Rican and Mexican subjects with asthma genotyped on the Affymetrix 6.0 GeneChip as part of the Genetics of Asthma in Latino Americans (GALA) study [20]. The third sample consists of 1,310 individuals from Mexico City participating in a type 2 diabetes study that were genotyped with the Affymetrix 5.0 GeneChip. The fourth sample contains 312 subjects in the Mexican Genome Diversity Project (MGDP) recruited by the National Institute of Genomic Medicine (INMEGEN) from throughout Mexico, including 48 subjects from Guanajuato, 50 subjects from Guerrero, 48 subjects from Sonora, 17 subjects from Tamaulipas, 50 subjects from Veracruz, 49 subjects from Yucatan, and 50 subjects from Zacatecas [30], [50]. A map of the geographic distribution of MGDP-INMEGEN samples is shown in Figure S2. A description of all the validation samples is shown in Table 6.

**Table 6:** Samples used for validation.

Population	Ethnicity	Sample size	Platform(s)
**GALA**	Mexican	668	Affymetrix 6.0
**GALA**	Puerto Rican	803	Affymetrix 6.0
**MGDP-INMEGEN**	Mexican	312	Affymetrix 500K+Illumina 550
**Mexico City**	Mexican	1310	Affymetrix 5.0

We implemented a three-population model to estimate individual ancestry proportions from genome-wide data using the program ADMIXTURE [57]. We filtered our genome-wide markers to eliminate markers in linkage disequilibrium at r^2^>0.8. Genotypes from ancestral populations described above defined the ancestral clusters relevant to Latin Americans. We also estimated ancestry using the panel of AIMs identified with the protocol above. The performance of the AIMs panel was established by calculating the correlation coefficient (R^2^) and measures of discordance (mean error, mean absolute error, and root mean squared error).

### Stage three: Genotyping of populations throughout Latin America

**Figure 6:**
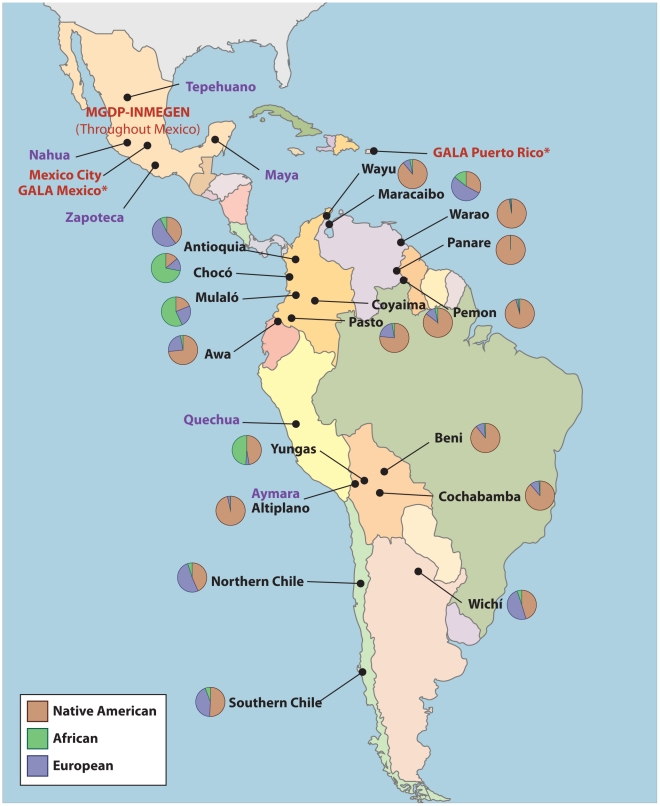
Origin of samples used in this study. Labels in purple correspond to the Native American ancestral populations, labels in red to the validation samples, and labels in black to the 18 populations from throughout the Americas. MGDP-INMEGEN samples were collected throughout Mexico (see Figure S1). GALA Mexico samples were also collected in the San Francisco Bay Area, CA. GALA Puerto Rico samples were also collected in New York, NY.

Using the validated AIMs panel, we genotyped 18 populations collected from Bolivia, Colombia, Venezuela, Argentina, and Chile. A description of the origin of the samples is provided in Text S1 and in Table 7 and Figure 6.

**Table 7:** Latin American populations genotyped in stage III of this study.

Population	Country	Ethnicity	Sample size
**Awa**	Colombia (Southern)	Indigenous	22
**Coyaima**	Colombia (Central)	Indigenous	19
**Pastos**	Colombia (Southern)	Indigenous	36
**Panare**	Venezuela (Amazon)	Indigenous	20
**Pemon**	Venezuela (Amazon)	Indigenous	20
**Warao**	Venezuela (Amazon)	Indigenous	20
**Wayu**	Venezuela (North)	Indigenous	20
**Wichi**	Argentina	Indigenous	14
**Maracaibo**	Venezuela	Mestizo (admixed)	20
**Northern Chile**	Chile	Mestizo (admixed)	20
**Southern Chile**	Chile	Mestizo (admixed)	20
**Antioquia**	Colombia	Mestizo (admixed)	19
**Antiplano**	Bolivia	Mestizo (admixed)	11
**Chocó**	Colombia	Afro-Colombian	35
**Mulaló**	Colombia	Afro-Colombian	28
**Beni**	Bolivia	Multi-ethnic (Mestizo and Indigenous)	10
**Cochabamba**	Bolivia	Multi-ethnic (Mestizo and Indigenous)	12
**Yungas**	Bolivia	Multi-ethnic (Indigenous, Afro-Bolivian)	27

### Genotyping

Subjects were genotyped on a Sequenom platform with the 400 most informative AIMs identified in phase I. AIMs were included in the final analysis if they had a genotyping call rate greater than 95% and Hardy-Weinberg equilibrium in each population individually. We required that all samples had genotyping missing data rates of <10%. Sample population groups were excluded if they have average genotyping data rates of <10%

### Software and statistical analysis

File merging, strand flipping, allele frequency determination, linkage disequilibrium calculations, and identity by descent estimations were performed with the program plink [52]. The algorithm to develop the panel of AIMs was implemented in Python version 2.6 [58]. Individual ancestral estimates were performed with a three-population model using a model-based likelihood estimation using the program ADMIXTURE [57]. Statistical analyses were performed with R and Python [58], [59]. Estimation of time since admixture was performed using the program ADMIXMAP [60], [61], [62], assuming an average of 29 years per generation [63].

### Web resources

Source code for the AIMs selection script is available at http://bts.ucsf.edu/burchard/.

## Supporting Information

Figure S1 QQ plots of genetic association studies of Diabetes in Mexicans, using nested sets of AIMs.
(PDF)

Figure S2 Origin of Mexican samples from MGDP-INMEGEN. Locations in purple correspond to Native American populations; those in red correspond to admixed MGDP-INMEGEN populations used for validation.
(PDF)

Table S1 AIMs used.
(DOCX)

Text S1 Supplemental methods, including detailed ethics statement and description of samples obtained for genotyping of populations throughout the Americas.
(DOCX)
