# Supplementary material for: Development of a Panel of Genome-Wide Ancestry Informative Markers to Study Admixture Throughout the Americas
Source: PLoS Genet. 2012 Mar 8;8(3):e1002554. doi: 10.1371/journal.pgen.1002554 (PMC3297575; doi:10.1371/journal.pgen.1002554)

No adjustment for stratification

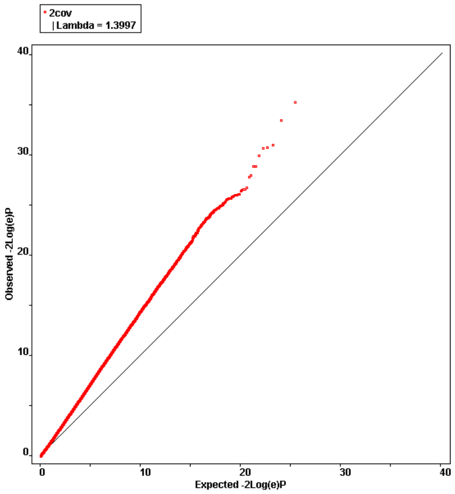

Adjustment using 22 AIMs

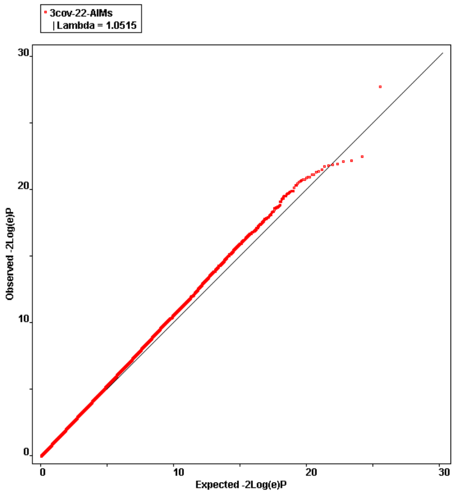

Adjustment using 41 AIMs

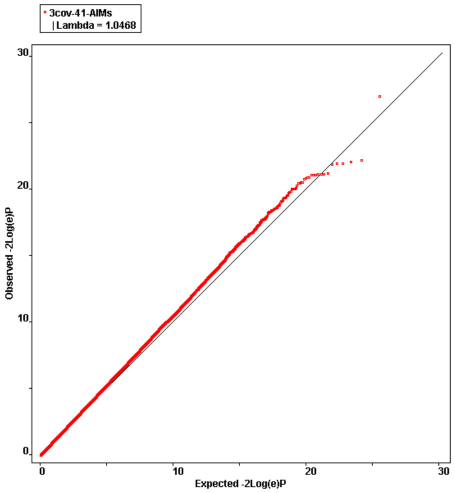

Adjustment using 82 AIMs

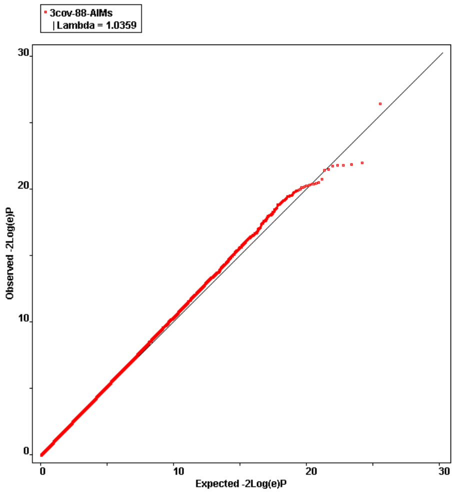

Adjustment using 194 AIMs

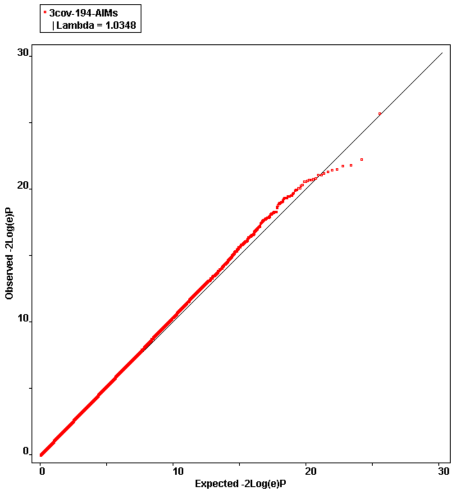

Adjustment using 314 AIMs

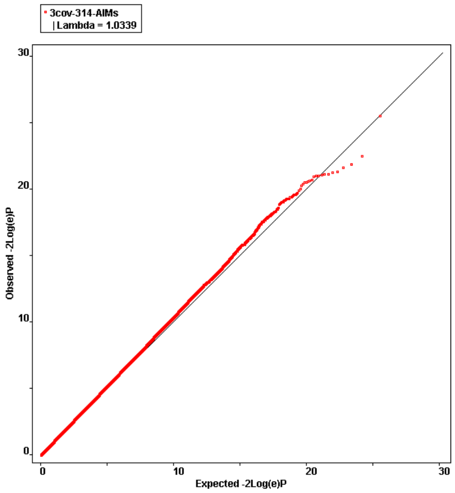

Supplement: Figure S1 — QQ plots of genetic association studies of Diabetes in Mexicans, using nested sets of AIMs. (PDF) [file pgen.1002554.s001.pdf]
