# Supplementary material for: Development of a Panel of Genome-Wide Ancestry Informative Markers to Study Admixture Throughout the Americas
Source: PLoS Genet. 2012 Mar 8;8(3):e1002554. doi: 10.1371/journal.pgen.1002554 (PMC3297575; doi:10.1371/journal.pgen.1002554)
Supplement: Figure S2 — Origin of Mexican samples from MGDP-INMEGEN. Locations in purple correspond to Native American populations; those in red correspond to admixed MGDP-INMEGEN populations used for validation. (PDF) [file pgen.1002554.s002.pdf]

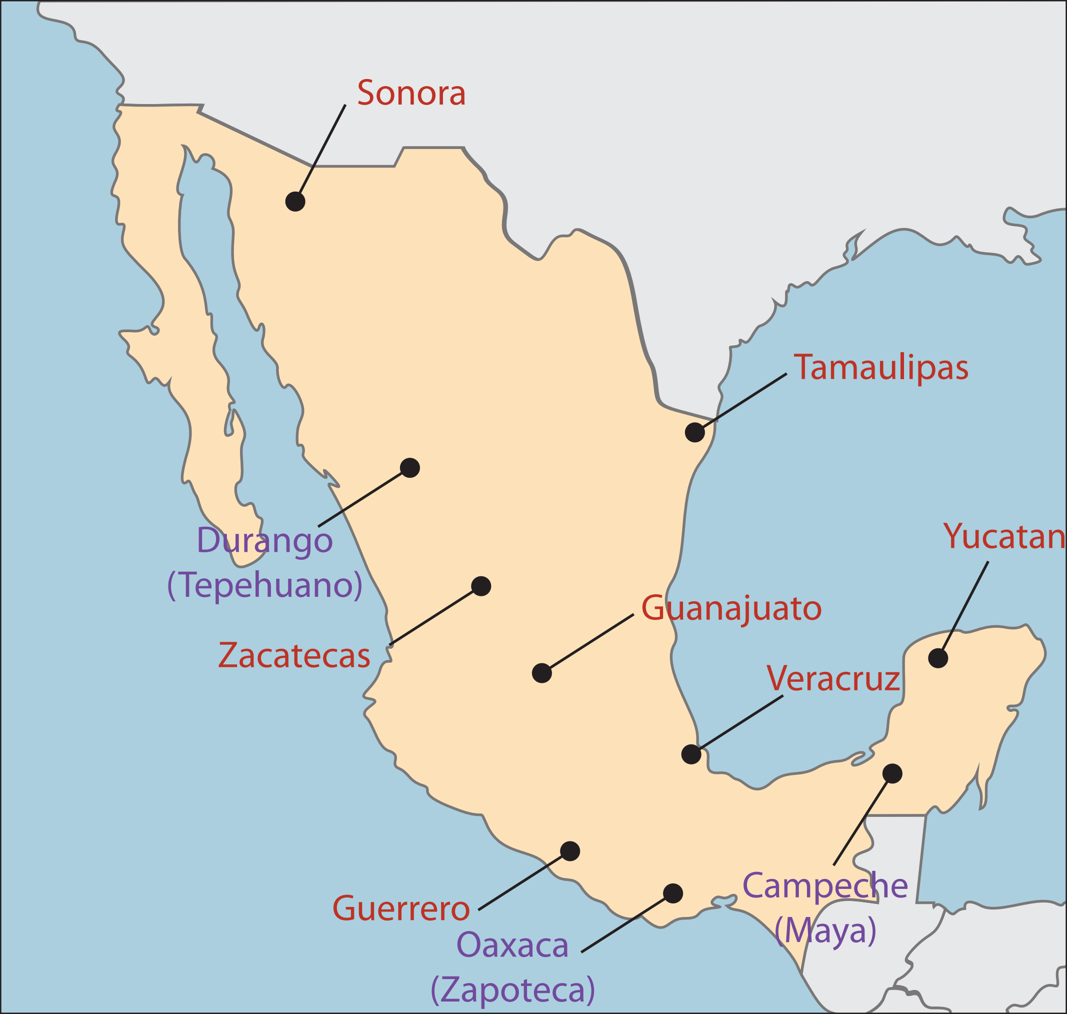
Sonora
Tamaulipas
Durango
(Tepehuano)
Yucatan
Guanajuato
Zacatecas
Veracruz
Campeche
(Maya)
Guerrero
Oaxaca
(Zapoteca)
