# Supplementary material for: Development of a Panel of Genome-Wide Ancestry Informative Markers to Study Admixture Throughout the Americas
Source: PLoS Genet. 2012 Mar 8;8(3):e1002554. doi: 10.1371/journal.pgen.1002554 (PMC3297575; doi:10.1371/journal.pgen.1002554)
Supplement: Table S1 — AIMs used. (DOCX) [file pgen.1002554.s003.docx]

# Supplemental Tables

Table S1: AIMs used.

| SNP rsID | chr | position | A1 | A2 | NAM_AF | EUR_AF | AFR_AF | population | LSBL(Fst) | LSBL(In) |
| --- | --- | --- | --- | --- | --- | --- | --- | --- | --- | --- |
| rs6685064 | 1 | 1201155 | C | T | 0.244 | 0.930 | 0.422 | EUR | 0.372 | 0.209 |
| rs12085319 | 1 | 10952065 | G | T | 0.347 | 0.817 | 0.015 | EUR | 0.351 | 0.205 |
| rs2745285 | 1 | 11549445 | G | A | 0.108 | 0.330 | 0.980 | AFR | 0.581 | 0.355 |
| rs4920310 | 1 | 18436476 | G | A | 0.305 | 0.819 | 0.117 | EUR | 0.355 | 0.194 |
| rs6684063 | 1 | 30471927 | T | G | 0.183 | 0.841 | 0.218 | EUR | 0.411 | 0.223 |
| rs7515867 | 1 | 31497331 | C | T | 0.062 | 0.613 | 0.039 | EUR | 0.356 | 0.202 |
| rs11264115 | 1 | 35429251 | G | A | 0.668 | 0.047 | 0.617 | EUR | 0.392 | 0.223 |
| rs11206160 | 1 | 53684400 | C | G | 0.015 | 0.200 | 0.922 | AFR | 0.633 | 0.381 |
| rs2984915 | 1 | 59026283 | T | C | 0.011 | 0.090 | 0.853 | AFR | 0.636 | 0.378 |
| rs6681578 | 1 | 64786174 | T | C | 0.080 | 0.233 | 0.947 | AFR | 0.616 | 0.364 |
| rs12743599 | 1 | 65140630 | C | T | 0.151 | 0.726 | 0.107 | EUR | 0.363 | 0.197 |
| rs12727814 | 1 | 71786781 | G | A | 0.057 | 0.752 | 0.078 | EUR | 0.484 | 0.273 |
| rs12022561 | 1 | 80008407 | T | C | 0.164 | 0.876 | 0.927 | NAM | 0.544 | 0.307 |
| rs6681719 | 1 | 112340266 | G | A | 0.054 | 0.895 | 0.733 | NAM | 0.575 | 0.336 |
| rs12065716 | 1 | 116575568 | G | C | 0.389 | 0.885 | 0.136 | EUR | 0.372 | 0.207 |
| rs12021830 | 1 | 119363905 | T | C | 0.107 | 0.838 | 0.995 | NAM | 0.626 | 0.376 |
| rs6693833 | 1 | 160209682 | C | T | 0.229 | 0.772 | 0.053 | EUR | 0.382 | 0.213 |
| rs12123239 | 1 | 196506434 | A | G | 0.023 | 0.608 | 0.015 | EUR | 0.403 | 0.241 |
| rs927900 | 1 | 199726581 | T | C | 0.043 | 0.620 | 0.053 | EUR | 0.367 | 0.208 |
| rs2025236 | 1 | 203049828 | A | G | 0.081 | 0.887 | 0.277 | EUR | 0.484 | 0.275 |
| rs1041310 | 1 | 203728158 | T | A | 0.118 | 0.839 | 0.340 | EUR | 0.355 | 0.195 |
| rs10779334 | 1 | 217173027 | G | A | 0.008 | 0.097 | 0.837 | AFR | 0.606 | 0.360 |
| rs12142968 | 1 | 232760664 | G | T | 0.081 | 0.736 | 0.888 | NAM | 0.530 | 0.303 |
| rs7525142 | 1 | 238809528 | T | G | 0.205 | 0.252 | 0.985 | AFR | 0.599 | 0.363 |
| rs9725312 | 1 | 243105441 | A | C | 0.000 | 0.060 | 0.843 | AFR | 0.659 | 0.400 |
| rs6677662 | 1 | 243557314 | G | A | 0.271 | 0.752 | 0.029 | EUR | 0.333 | 0.190 |
| rs7527770 | 1 | 246293824 | A | C | 0.019 | 0.173 | 0.941 | AFR | 0.690 | 0.419 |
| rs41009 | 2 | 8012609 | A | G | 0.011 | 0.154 | 0.879 | AFR | 0.610 | 0.362 |
| rs181130 | 2 | 8190471 | C | G | 0.179 | 0.868 | 0.981 | NAM | 0.544 | 0.317 |
| rs2140701 | 2 | 9725153 | G | A | 0.034 | 0.586 | 0.937 | NAM | 0.501 | 0.307 |
| rs1367158 | 2 | 16454446 | G | A | 0.080 | 0.720 | 0.189 | EUR | 0.343 | 0.187 |
| rs798364 | 2 | 16762671 | A | G | 0.008 | 0.192 | 0.927 | AFR | 0.652 | 0.396 |
| rs2710684 | 2 | 17677511 | C | T | 0.065 | 0.783 | 0.854 | NAM | 0.573 | 0.329 |
| rs6711746 | 2 | 29292053 | T | C | 0.102 | 0.809 | 0.874 | NAM | 0.546 | 0.308 |
| rs10175357 | 2 | 35015176 | C | T | 0.038 | 0.059 | 0.820 | AFR | 0.605 | 0.352 |
| rs305163 | 2 | 36163166 | G | A | 0.172 | 0.712 | 0.063 | EUR | 0.356 | 0.196 |
| rs11124754 | 2 | 40729617 | T | C | 0.213 | 0.919 | 0.296 | EUR | 0.452 | 0.252 |
| rs10179648 | 2 | 43661569 | T | C | 0.076 | 0.686 | 0.966 | NAM | 0.526 | 0.313 |
| rs6740875 | 2 | 44971206 | G | A | 0.321 | 0.848 | 0.175 | EUR | 0.355 | 0.193 |
| rs2595214 | 2 | 46071174 | A | G | 0.168 | 0.852 | 1.000 | NAM | 0.550 | 0.328 |
| rs4643526 | 2 | 61038155 | A | G | 0.019 | 0.177 | 0.917 | AFR | 0.646 | 0.387 |
| rs4671675 | 2 | 65715159 | T | C | 0.298 | 0.843 | 0.194 | EUR | 0.355 | 0.191 |
| rs7584385 | 2 | 69094763 | A | G | 0.206 | 0.806 | 0.078 | EUR | 0.432 | 0.240 |
| rs10496176 | 2 | 70027133 | T | C | 0.061 | 0.821 | 0.835 | NAM | 0.595 | 0.342 |
| rs7601949 | 2 | 79882679 | C | T | 0.027 | 0.523 | 0.010 | EUR | 0.321 | 0.192 |
| rs12328713 | 2 | 84022392 | T | A | 0.374 | 0.948 | 0.441 | EUR | 0.334 | 0.188 |
| rs7594727 | 2 | 96853597 | A | C | 0.008 | 0.136 | 0.869 | AFR | 0.615 | 0.366 |
| rs1257084 | 2 | 97121236 | T | C | 0.212 | 0.768 | 0.068 | EUR | 0.385 | 0.213 |
| rs7598069 | 2 | 98127823 | G | A | 0.011 | 0.610 | 0.971 | NAM | 0.571 | 0.368 |
| rs1567803 | 2 | 100709450 | T | C | 0.019 | 0.642 | 0.029 | EUR | 0.429 | 0.253 |
| rs260690 | 2 | 108946170 | A | C | 0.078 | 0.935 | 0.384 | EUR | 0.470 | 0.277 |
| rs6431064 | 2 | 129676012 | C | A | 0.088 | 0.587 | 0.015 | EUR | 0.320 | 0.185 |
| rs1036543 | 2 | 133392684 | A | G | 0.282 | 0.939 | 0.350 | EUR | 0.413 | 0.233 |
| rs7589619 | 2 | 143001328 | C | T | 0.050 | 0.218 | 0.922 | AFR | 0.604 | 0.355 |
| rs6429990 | 2 | 143365273 | G | C | 0.023 | 0.570 | 0.024 | EUR | 0.358 | 0.211 |
| rs1196705 | 2 | 149927468 | G | A | 0.015 | 0.638 | 0.117 | EUR | 0.344 | 0.198 |
| rs298259 | 2 | 157033071 | C | T | 0.160 | 0.761 | 0.131 | EUR | 0.381 | 0.206 |
| rs10497191 | 2 | 158375463 | T | C | 0.115 | 0.151 | 0.951 | AFR | 0.674 | 0.397 |
| rs6759948 | 2 | 158597839 | A | G | 0.011 | 0.599 | 0.087 | EUR | 0.334 | 0.194 |
| rs6759814 | 2 | 163101007 | T | C | 0.263 | 0.945 | 0.296 | EUR | 0.466 | 0.265 |
| rs1809119 | 2 | 174234175 | C | A | 0.046 | 0.207 | 0.917 | AFR | 0.608 | 0.357 |
| rs2044770 | 2 | 174892151 | A | G | 0.076 | 0.824 | 0.971 | NAM | 0.654 | 0.390 |
| rs1005932 | 2 | 177039956 | A | G | 0.038 | 0.664 | 0.922 | NAM | 0.556 | 0.334 |
| rs3927978 | 2 | 178152100 | G | A | 0.027 | 0.288 | 0.990 | AFR | 0.667 | 0.429 |
| rs2695735 | 2 | 178496734 | A | G | 0.023 | 0.609 | 0.902 | NAM | 0.529 | 0.324 |
| rs7568054 | 2 | 190206141 | G | A | 0.053 | 0.651 | 0.005 | EUR | 0.422 | 0.251 |
| rs6707773 | 2 | 191052377 | C | T | 0.057 | 0.764 | 0.791 | NAM | 0.533 | 0.305 |
| rs6710083 | 2 | 192480837 | T | G | 0.004 | 0.078 | 0.840 | AFR | 0.633 | 0.380 |
| rs1606237 | 2 | 205955695 | T | C | 0.019 | 0.334 | 0.971 | AFR | 0.591 | 0.371 |
| rs16851773 | 2 | 214897054 | G | A | 0.126 | 0.647 | 0.024 | EUR | 0.342 | 0.195 |
| rs10186877 | 2 | 216327063 | C | G | 0.103 | 0.709 | 0.058 | EUR | 0.410 | 0.229 |
| rs1472714 | 3 | 1067542 | G | A | 0.027 | 0.614 | 0.040 | EUR | 0.385 | 0.223 |
| rs4109078 | 3 | 3215356 | T | C | 0.111 | 0.858 | 0.903 | NAM | 0.591 | 0.336 |
| rs6768750 | 3 | 7215675 | G | A | 0.073 | 0.695 | 0.976 | NAM | 0.542 | 0.325 |
| rs1605524 | 3 | 21351652 | G | A | 0.080 | 0.915 | 0.471 | EUR | 0.369 | 0.215 |
| rs9863982 | 3 | 31509158 | C | T | 0.008 | 0.234 | 0.942 | AFR | 0.636 | 0.390 |
| rs10222597 | 3 | 33239918 | C | T | 0.195 | 0.914 | 0.961 | NAM | 0.559 | 0.321 |
| rs2282751 | 3 | 50266789 | G | A | 0.248 | 0.870 | 0.189 | EUR | 0.426 | 0.233 |
| rs4955903 | 3 | 54884236 | T | C | 0.023 | 0.647 | 0.956 | NAM | 0.579 | 0.362 |
| rs978979 | 3 | 56508056 | G | A | 0.027 | 0.650 | 0.092 | EUR | 0.374 | 0.213 |
| rs6803290 | 3 | 59667208 | C | T | 0.023 | 0.636 | 0.010 | EUR | 0.435 | 0.262 |
| rs862500 | 3 | 64247689 | A | T | 0.134 | 0.821 | 0.117 | EUR | 0.485 | 0.268 |
| rs9833943 | 3 | 65091717 | A | G | 0.069 | 0.691 | 0.971 | NAM | 0.544 | 0.326 |
| rs783511 | 3 | 66558558 | C | T | 0.147 | 0.662 | 0.044 | EUR | 0.331 | 0.185 |
| rs3925004 | 3 | 69046190 | C | A | 0.115 | 0.807 | 0.956 | NAM | 0.570 | 0.330 |
| rs13097560 | 3 | 85212019 | A | G | 0.046 | 0.658 | 0.068 | EUR | 0.392 | 0.221 |
| rs7644167 | 3 | 98043206 | C | T | 0.351 | 0.921 | 0.296 | EUR | 0.378 | 0.209 |
| rs2317212 | 3 | 98810974 | G | T | 0.073 | 0.878 | 0.340 | EUR | 0.422 | 0.240 |
| rs7630522 | 3 | 108635778 | C | T | 0.058 | 0.787 | 0.262 | EUR | 0.372 | 0.208 |
| rs4855697 | 3 | 110349818 | A | G | 0.011 | 0.597 | 0.913 | NAM | 0.543 | 0.342 |
| rs7630111 | 3 | 115648591 | A | C | 0.186 | 0.756 | 0.117 | EUR | 0.366 | 0.198 |
| rs2869782 | 3 | 118567115 | T | C | 0.023 | 0.053 | 0.811 | AFR | 0.609 | 0.358 |
| rs2937673 | 3 | 118715077 | G | A | 0.019 | 0.553 | 0.044 | EUR | 0.327 | 0.190 |
| rs16832787 | 3 | 123402430 | G | A | 0.095 | 0.823 | 0.947 | NAM | 0.611 | 0.355 |
| rs12638324 | 3 | 124089788 | G | A | 0.137 | 0.893 | 0.903 | NAM | 0.579 | 0.328 |
| rs6791346 | 3 | 124245130 | T | C | 0.000 | 0.151 | 0.917 | AFR | 0.678 | 0.414 |
| rs820371 | 3 | 124887401 | G | A | 0.258 | 0.792 | 0.044 | EUR | 0.386 | 0.218 |
| rs2855557 | 3 | 130734612 | T | A | 0.275 | 0.151 | 0.971 | AFR | 0.588 | 0.349 |
| rs1586861 | 3 | 140541186 | T | C | 0.046 | 0.899 | 0.863 | NAM | 0.700 | 0.415 |
| rs868767 | 3 | 142860020 | A | G | 0.149 | 0.894 | 0.981 | NAM | 0.614 | 0.361 |
| rs9832471 | 3 | 145053561 | C | A | 0.008 | 0.057 | 0.811 | AFR | 0.613 | 0.366 |
| rs2106124 | 3 | 170540703 | G | A | 0.031 | 0.739 | 0.155 | EUR | 0.414 | 0.235 |
| rs6806083 | 3 | 184287079 | A | G | 0.267 | 0.746 | 0.005 | EUR | 0.334 | 0.195 |
| rs6770338 | 3 | 187393301 | A | G | 0.093 | 0.399 | 0.990 | AFR | 0.549 | 0.346 |
| rs2378269 | 3 | 188705920 | T | G | 0.069 | 0.659 | 0.951 | NAM | 0.510 | 0.303 |
| rs13327370 | 3 | 199228595 | G | A | 0.011 | 0.230 | 0.922 | AFR | 0.606 | 0.365 |
| rs7665516 | 4 | 11243690 | G | A | 0.023 | 0.637 | 0.888 | NAM | 0.546 | 0.333 |
| rs719776 | 4 | 33363055 | C | G | 0.225 | 0.119 | 0.961 | AFR | 0.628 | 0.371 |
| rs6817183 | 4 | 34174660 | A | C | 0.141 | 0.168 | 0.937 | AFR | 0.616 | 0.356 |
| rs11725412 | 4 | 37954149 | G | A | 0.050 | 0.931 | 0.816 | NAM | 0.672 | 0.400 |
| rs10026397 | 4 | 41260644 | A | G | 0.046 | 0.194 | 0.932 | AFR | 0.643 | 0.381 |
| rs11051 | 4 | 41783934 | G | A | 0.080 | 0.262 | 0.971 | AFR | 0.635 | 0.384 |
| rs12650513 | 4 | 61243531 | G | A | 0.235 | 0.958 | 0.976 | NAM | 0.557 | 0.327 |
| rs1486498 | 4 | 61554674 | C | T | 0.031 | 0.561 | 0.024 | EUR | 0.342 | 0.200 |
| rs6855458 | 4 | 79829056 | A | G | 0.134 | 0.654 | 0.035 | EUR | 0.339 | 0.190 |
| rs4693573 | 4 | 84318348 | C | T | 0.011 | 0.171 | 0.888 | AFR | 0.608 | 0.362 |
| rs17014118 | 4 | 89538319 | T | C | 0.317 | 0.848 | 0.155 | EUR | 0.366 | 0.200 |
| rs2089990 | 4 | 93519211 | A | C | 0.081 | 0.819 | 0.947 | NAM | 0.631 | 0.370 |
| rs2851060 | 4 | 102172345 | T | C | 0.073 | 0.771 | 0.898 | NAM | 0.576 | 0.332 |
| rs920559 | 4 | 102437738 | C | G | 0.275 | 0.856 | 0.211 | EUR | 0.378 | 0.205 |
| rs6533426 | 4 | 110512886 | G | A | 0.012 | 0.147 | 0.859 | AFR | 0.587 | 0.347 |
| rs4541508 | 4 | 110901936 | C | T | 0.134 | 0.680 | 0.083 | EUR | 0.341 | 0.186 |
| rs6533531 | 4 | 111951414 | T | G | 0.050 | 0.599 | 0.034 | EUR | 0.356 | 0.203 |
| rs7664927 | 4 | 114277822 | A | G | 0.107 | 0.950 | 0.820 | NAM | 0.592 | 0.343 |
| rs17015014 | 4 | 142948222 | G | C | 0.115 | 0.869 | 0.947 | NAM | 0.623 | 0.360 |
| rs1391099 | 4 | 143593360 | A | G | 0.174 | 0.848 | 0.291 | EUR | 0.376 | 0.204 |
| rs924780 | 4 | 148905447 | A | C | 0.115 | 0.190 | 0.942 | AFR | 0.625 | 0.364 |
| rs6835367 | 4 | 151729289 | T | C | 0.008 | 0.104 | 0.884 | AFR | 0.670 | 0.402 |
| rs1155422 | 4 | 156628123 | T | G | 0.012 | 0.505 | 0.015 | EUR | 0.315 | 0.190 |
| rs7664076 | 4 | 172625303 | A | G | 0.344 | 0.803 | 0.034 | EUR | 0.334 | 0.191 |
| rs12715977 | 4 | 177732408 | C | A | 0.145 | 0.752 | 0.170 | EUR | 0.356 | 0.191 |
| rs17090615 | 4 | 178982610 | T | C | 0.374 | 0.880 | 0.218 | EUR | 0.344 | 0.187 |
| rs3911610 | 4 | 180709168 | C | T | 0.347 | 0.936 | 0.388 | EUR | 0.355 | 0.198 |
| rs10071261 | 5 | 1066694 | T | C | 0.107 | 0.086 | 0.903 | AFR | 0.650 | 0.375 |
| rs378257 | 5 | 2571738 | T | C | 0.035 | 0.253 | 0.942 | AFR | 0.610 | 0.366 |
| rs1875017 | 5 | 10504154 | A | G | 0.286 | 0.894 | 0.301 | EUR | 0.374 | 0.204 |
| rs10042283 | 5 | 15968161 | T | G | 0.069 | 0.663 | 0.044 | EUR | 0.399 | 0.226 |
| rs10068162 | 5 | 22602971 | T | C | 0.206 | 0.876 | 0.995 | NAM | 0.521 | 0.308 |
| rs10461813 | 5 | 24659860 | A | G | 0.046 | 0.237 | 0.951 | AFR | 0.637 | 0.383 |
| rs4867557 | 5 | 28496197 | C | T | 0.126 | 0.761 | 0.199 | EUR | 0.357 | 0.193 |
| rs1946267 | 5 | 33957567 | G | A | 0.252 | 0.793 | 0.097 | EUR | 0.371 | 0.203 |
| rs353373 | 5 | 40261722 | G | A | 0.191 | 0.863 | 1.000 | NAM | 0.530 | 0.315 |
| rs349323 | 5 | 100804840 | T | A | 0.031 | 0.629 | 0.015 | EUR | 0.417 | 0.247 |
| rs6866970 | 5 | 100978603 | G | A | 0.015 | 0.060 | 0.858 | AFR | 0.674 | 0.403 |
| rs17157450 | 5 | 104384818 | C | T | 0.145 | 0.864 | 0.903 | NAM | 0.544 | 0.305 |
| rs13354224 | 5 | 108848634 | C | T | 0.008 | 0.111 | 0.903 | AFR | 0.693 | 0.419 |
| rs509237 | 5 | 112578146 | G | A | 0.191 | 0.794 | 0.039 | EUR | 0.447 | 0.254 |
| rs10079352 | 5 | 117522539 | A | G | 0.031 | 0.597 | 0.961 | NAM | 0.523 | 0.325 |
| rs2227282 | 5 | 132041078 | G | C | 0.103 | 0.730 | 0.005 | EUR | 0.461 | 0.271 |
| rs7732591 | 5 | 142105361 | C | T | 0.313 | 0.148 | 0.990 | AFR | 0.595 | 0.368 |
| rs4145160 | 5 | 152854742 | G | A | 0.127 | 0.953 | 0.850 | NAM | 0.589 | 0.340 |
| rs1366220 | 5 | 153477973 | C | T | 0.038 | 0.691 | 0.947 | NAM | 0.588 | 0.357 |
| rs11960137 | 5 | 155270659 | C | G | 0.122 | 0.875 | 0.942 | NAM | 0.614 | 0.353 |
| rs1107154 | 5 | 165319891 | T | C | 0.061 | 0.390 | 0.985 | AFR | 0.557 | 0.352 |
| rs11743823 | 5 | 168097322 | C | T | 0.103 | 0.814 | 0.947 | NAM | 0.590 | 0.342 |
| rs2042314 | 5 | 170110158 | G | A | 0.061 | 0.860 | 0.854 | NAM | 0.638 | 0.370 |
| rs6875659 | 5 | 175091259 | A | G | 0.038 | 0.095 | 0.942 | AFR | 0.761 | 0.460 |
| rs17648108 | 5 | 177764162 | T | C | 0.042 | 0.755 | 0.204 | EUR | 0.386 | 0.217 |
| rs860747 | 6 | 14808928 | A | G | 0.008 | 0.152 | 0.937 | AFR | 0.709 | 0.434 |
| rs2754777 | 6 | 24898441 | A | C | 0.027 | 0.516 | 0.005 | EUR | 0.317 | 0.192 |
| rs9357427 | 6 | 44085513 | G | A | 0.057 | 0.202 | 0.917 | AFR | 0.606 | 0.354 |
| rs10498810 | 6 | 56234862 | C | A | 0.079 | 0.887 | 0.947 | NAM | 0.698 | 0.411 |
| rs1325476 | 6 | 84598672 | T | C | 0.424 | 0.921 | 0.165 | EUR | 0.388 | 0.220 |
| rs794672 | 6 | 95515038 | A | G | 0.112 | 0.142 | 0.922 | AFR | 0.634 | 0.365 |
| rs7752055 | 6 | 106067405 | C | T | 0.038 | 0.119 | 0.893 | AFR | 0.656 | 0.384 |
| rs2503770 | 6 | 110373108 | A | T | 0.221 | 0.765 | 0.102 | EUR | 0.358 | 0.195 |
| rs9384878 | 6 | 114081811 | C | T | 0.015 | 0.649 | 0.848 | NAM | 0.553 | 0.338 |
| rs218862 | 6 | 121425761 | C | T | 0.153 | 0.837 | 0.097 | EUR | 0.506 | 0.282 |
| rs512342 | 6 | 125441391 | G | C | 0.023 | 0.667 | 0.947 | NAM | 0.593 | 0.368 |
| rs9689446 | 6 | 134503136 | C | T | 0.008 | 0.059 | 0.811 | AFR | 0.611 | 0.365 |
| rs9321456 | 6 | 134828954 | T | C | 0.023 | 0.660 | 0.160 | EUR | 0.327 | 0.186 |
| rs7750326 | 6 | 145079271 | A | G | 0.038 | 0.746 | 0.078 | EUR | 0.490 | 0.280 |
| rs2065664 | 6 | 150125314 | T | C | 0.039 | 0.639 | 0.099 | EUR | 0.350 | 0.197 |
| rs4598087 | 6 | 153966561 | G | A | 0.027 | 0.241 | 0.932 | AFR | 0.608 | 0.364 |
| rs10946113 | 6 | 158504679 | C | A | 0.151 | 0.896 | 0.971 | NAM | 0.607 | 0.354 |
| rs9457490 | 6 | 159219204 | C | T | 0.096 | 0.723 | 0.966 | NAM | 0.527 | 0.310 |
| rs7771341 | 6 | 162006932 | G | C | 0.149 | 0.851 | 0.985 | NAM | 0.572 | 0.336 |
| rs6605534 | 6 | 169464466 | G | A | 0.058 | 0.855 | 0.874 | NAM | 0.654 | 0.381 |
| rs7769343 | 6 | 170359831 | T | G | 0.008 | 0.099 | 0.840 | AFR | 0.610 | 0.362 |
| rs12701745 | 7 | 4558764 | G | A | 0.012 | 0.526 | 0.024 | EUR | 0.325 | 0.194 |
| rs731257 | 7 | 12635776 | G | A | 0.160 | 0.880 | 0.976 | NAM | 0.581 | 0.339 |
| rs2080161 | 7 | 13297675 | A | C | 0.099 | 0.798 | 0.976 | NAM | 0.593 | 0.351 |
| rs7808899 | 7 | 14779699 | T | G | 0.298 | 0.830 | 0.146 | EUR | 0.362 | 0.197 |
| rs2106362 | 7 | 18691786 | C | G | 0.287 | 0.819 | 0.132 | EUR | 0.362 | 0.197 |
| rs7794503 | 7 | 31015423 | C | T | 0.008 | 0.228 | 0.961 | AFR | 0.675 | 0.420 |
| rs17170516 | 7 | 32785284 | G | A | 0.164 | 0.841 | 0.981 | NAM | 0.540 | 0.315 |
| rs2598391 | 7 | 33589891 | A | G | 0.004 | 0.300 | 0.961 | AFR | 0.608 | 0.380 |
| rs37268 | 7 | 41305505 | C | T | 0.103 | 0.222 | 0.942 | AFR | 0.606 | 0.353 |
| rs259162 | 7 | 89466838 | A | C | 0.127 | 0.696 | 0.113 | EUR | 0.344 | 0.186 |
| rs11763970 | 7 | 97655257 | G | A | 0.034 | 0.547 | 0.024 | EUR | 0.327 | 0.190 |
| rs2384982 | 7 | 103493636 | C | T | 0.187 | 0.938 | 0.398 | EUR | 0.424 | 0.242 |
| rs7797239 | 7 | 117476459 | C | T | 0.308 | 0.826 | 0.141 | EUR | 0.352 | 0.191 |
| rs2299542 | 7 | 126573092 | A | G | 0.031 | 0.644 | 0.864 | NAM | 0.529 | 0.316 |
| rs350655 | 7 | 130364241 | A | C | 0.011 | 0.097 | 0.888 | AFR | 0.683 | 0.410 |
| rs2909680 | 7 | 130987063 | G | A | 0.260 | 0.865 | 0.209 | EUR | 0.401 | 0.218 |
| rs10258063 | 7 | 131478822 | A | G | 0.004 | 0.075 | 0.917 | AFR | 0.758 | 0.466 |
| rs2774969 | 7 | 138284445 | T | C | 0.058 | 0.355 | 0.995 | AFR | 0.606 | 0.391 |
| rs6605559 | 7 | 139070502 | A | G | 0.466 | 0.952 | 0.374 | EUR | 0.326 | 0.185 |
| rs344470 | 7 | 146044430 | G | A | 0.023 | 0.154 | 0.869 | AFR | 0.591 | 0.346 |
| rs2707575 | 7 | 147269656 | G | A | 0.031 | 0.574 | 0.034 | EUR | 0.348 | 0.201 |
| rs11784513 | 8 | 305843 | T | C | 0.130 | 0.174 | 0.932 | AFR | 0.612 | 0.352 |
| rs2924720 | 8 | 4827409 | A | G | 0.054 | 0.695 | 0.888 | NAM | 0.540 | 0.316 |
| rs366178 | 8 | 8808564 | G | T | 0.031 | 0.742 | 0.850 | NAM | 0.598 | 0.355 |
| rs17150066 | 8 | 9347921 | G | A | 0.088 | 0.858 | 0.985 | NAM | 0.674 | 0.405 |
| rs2736340 | 8 | 11381382 | C | T | 0.089 | 0.792 | 0.879 | NAM | 0.556 | 0.315 |
| rs11778591 | 8 | 12764720 | A | C | 0.057 | 0.908 | 0.418 | EUR | 0.407 | 0.238 |
| rs3810776 | 8 | 15663500 | A | G | 0.092 | 0.798 | 0.874 | NAM | 0.553 | 0.313 |
| rs6586662 | 8 | 17757470 | A | T | 0.103 | 0.724 | 0.151 | EUR | 0.363 | 0.197 |
| rs4871930 | 8 | 24747330 | G | A | 0.023 | 0.249 | 0.922 | AFR | 0.585 | 0.350 |
| rs1436612 | 8 | 35648583 | C | G | 0.011 | 0.178 | 0.898 | AFR | 0.617 | 0.368 |
| rs569688 | 8 | 61124375 | C | A | 0.096 | 0.811 | 0.917 | NAM | 0.583 | 0.334 |
| rs956969 | 8 | 62227592 | T | C | 0.034 | 0.623 | 0.049 | EUR | 0.381 | 0.218 |
| rs16880715 | 8 | 111524781 | T | G | 0.019 | 0.091 | 0.859 | AFR | 0.641 | 0.379 |
| rs13280988 | 8 | 112439692 | A | G | 0.031 | 0.581 | 0.058 | EUR | 0.333 | 0.190 |
| rs9642819 | 8 | 114231199 | G | A | 0.031 | 0.731 | 0.806 | NAM | 0.565 | 0.333 |
| rs6994396 | 8 | 114881166 | G | T | 0.115 | 0.865 | 0.850 | NAM | 0.552 | 0.310 |
| rs7017679 | 8 | 115827888 | G | A | 0.229 | 0.908 | 0.398 | EUR | 0.361 | 0.200 |
| rs799889 | 8 | 117320076 | T | G | 0.103 | 0.830 | 0.113 | EUR | 0.524 | 0.292 |
| rs2450219 | 8 | 119372638 | T | C | 0.057 | 0.641 | 0.058 | EUR | 0.374 | 0.210 |
| rs1357038 | 8 | 119771453 | A | T | 0.000 | 0.161 | 0.874 | AFR | 0.599 | 0.360 |
| rs7006331 | 8 | 123503731 | C | T | 0.050 | 0.599 | 0.049 | EUR | 0.346 | 0.195 |
| rs1876201 | 8 | 140052179 | G | A | 0.095 | 0.770 | 0.151 | EUR | 0.421 | 0.231 |
| rs10111852 | 8 | 142006723 | T | C | 0.023 | 0.590 | 0.015 | EUR | 0.385 | 0.229 |
| rs7048037 | 9 | 533590 | G | C | 0.065 | 0.685 | 0.121 | EUR | 0.365 | 0.202 |
| rs1500318 | 9 | 8351445 | T | C | 0.015 | 0.137 | 0.854 | AFR | 0.589 | 0.347 |
| rs1535661 | 9 | 10359368 | T | A | 0.038 | 0.609 | 0.083 | EUR | 0.334 | 0.188 |
| rs10756267 | 9 | 11701459 | G | A | 0.118 | 0.888 | 0.990 | NAM | 0.658 | 0.394 |
| rs791654 | 9 | 12480247 | A | G | 0.168 | 0.885 | 0.376 | EUR | 0.370 | 0.204 |
| rs10810942 | 9 | 18270143 | G | A | 0.053 | 0.846 | 0.794 | NAM | 0.596 | 0.345 |
| rs10811305 | 9 | 20047787 | G | C | 0.062 | 0.850 | 0.976 | NAM | 0.706 | 0.427 |
| rs3780827 | 9 | 20341419 | C | G | 0.618 | 0.077 | 0.704 | EUR | 0.364 | 0.201 |
| rs7864029 | 9 | 21920147 | G | C | 0.242 | 0.824 | 0.170 | EUR | 0.380 | 0.205 |
| rs1231375 | 9 | 25745952 | C | T | 0.244 | 0.852 | 0.243 | EUR | 0.374 | 0.201 |
| rs12380601 | 9 | 73838451 | A | G | 0.031 | 0.583 | 0.029 | EUR | 0.360 | 0.210 |
| rs7851160 | 9 | 79438617 | A | G | 0.191 | 0.837 | 0.172 | EUR | 0.430 | 0.234 |
| rs4146171 | 9 | 83122797 | C | T | 0.039 | 0.696 | 0.985 | NAM | 0.603 | 0.374 |
| rs4877824 | 9 | 86045679 | A | G | 0.069 | 0.614 | 0.058 | EUR | 0.338 | 0.189 |
| rs10746839 | 9 | 90073450 | G | A | 0.023 | 0.605 | 0.097 | EUR | 0.326 | 0.186 |
| rs1980889 | 9 | 91089065 | T | C | 0.107 | 0.935 | 0.961 | NAM | 0.709 | 0.420 |
| rs3001115 | 9 | 112443667 | C | T | 0.038 | 0.791 | 0.929 | NAM | 0.669 | 0.402 |
| rs10981899 | 9 | 115518226 | G | T | 0.181 | 0.897 | 0.985 | NAM | 0.573 | 0.337 |
| rs2809393 | 9 | 119931458 | C | T | 0.103 | 0.867 | 0.350 | EUR | 0.389 | 0.218 |
| rs10751867 | 10 | 2339064 | T | C | 0.351 | 0.916 | 0.175 | EUR | 0.429 | 0.241 |
| rs4881046 | 10 | 3023127 | T | A | 0.015 | 0.118 | 0.878 | AFR | 0.644 | 0.382 |
| rs907687 | 10 | 5940768 | G | T | 0.062 | 0.670 | 0.913 | NAM | 0.517 | 0.303 |
| rs4747860 | 10 | 10580170 | T | C | 0.080 | 0.689 | 0.131 | EUR | 0.353 | 0.193 |
| rs1326207 | 10 | 15206911 | C | T | 0.059 | 0.257 | 0.985 | AFR | 0.676 | 0.422 |
| rs4749305 | 10 | 28431602 | A | G | 0.135 | 0.859 | 0.345 | EUR | 0.370 | 0.204 |
| rs3123687 | 10 | 31569267 | C | G | 0.027 | 0.072 | 0.966 | AFR | 0.835 | 0.520 |
| rs4948800 | 10 | 43791290 | C | T | 0.046 | 0.590 | 0.063 | EUR | 0.327 | 0.184 |
| rs709616 | 10 | 48251333 | A | G | 0.027 | 0.071 | 0.864 | AFR | 0.665 | 0.393 |
| rs3810947 | 10 | 50491249 | G | A | 0.202 | 0.946 | 0.378 | EUR | 0.445 | 0.255 |
| rs2795918 | 10 | 55847021 | T | C | 0.046 | 0.692 | 0.850 | NAM | 0.533 | 0.311 |
| rs7911953 | 10 | 61461045 | T | G | 0.157 | 0.906 | 0.884 | NAM | 0.546 | 0.307 |
| rs10997469 | 10 | 68416291 | C | T | 0.137 | 0.787 | 0.985 | NAM | 0.528 | 0.310 |
| rs11000445 | 10 | 74342405 | C | T | 0.011 | 0.056 | 0.820 | AFR | 0.625 | 0.373 |
| rs7080350 | 10 | 75247849 | A | G | 0.027 | 0.700 | 0.165 | EUR | 0.363 | 0.206 |
| rs7097617 | 10 | 77174293 | G | A | 0.076 | 0.194 | 0.966 | AFR | 0.687 | 0.414 |
| rs7902158 | 10 | 83713437 | A | G | 0.047 | 0.763 | 0.039 | EUR | 0.539 | 0.312 |
| rs2245251 | 10 | 93050437 | T | C | 0.038 | 0.663 | 0.871 | NAM | 0.534 | 0.316 |
| rs11186960 | 10 | 94132587 | A | C | 0.008 | 0.564 | 0.039 | EUR | 0.348 | 0.208 |
| rs4244304 | 10 | 94926318 | C | T | 0.164 | 0.897 | 0.981 | NAM | 0.595 | 0.349 |
| rs4918326 | 10 | 109607547 | C | T | 0.061 | 0.738 | 0.947 | NAM | 0.590 | 0.350 |
| rs17130385 | 10 | 115186009 | G | T | 0.109 | 0.933 | 0.971 | NAM | 0.710 | 0.423 |
| rs7087634 | 10 | 118161006 | T | C | 0.313 | 0.901 | 0.136 | EUR | 0.452 | 0.254 |
| rs1419138 | 10 | 119721271 | A | G | 0.309 | 0.919 | 0.112 | EUR | 0.492 | 0.282 |
| rs12358575 | 10 | 122952924 | T | C | 0.107 | 0.724 | 0.131 | EUR | 0.375 | 0.204 |
| rs9422913 | 10 | 127335597 | C | T | 0.771 | 0.118 | 0.718 | EUR | 0.399 | 0.218 |
| rs10764919 | 10 | 131553641 | A | G | 0.046 | 0.570 | 0.019 | EUR | 0.340 | 0.198 |
| rs1352239 | 11 | 11322476 | T | G | 0.058 | 0.602 | 0.029 | EUR | 0.355 | 0.203 |
| rs10741584 | 11 | 12340445 | G | A | 0.023 | 0.133 | 0.879 | AFR | 0.627 | 0.369 |
| rs874189 | 11 | 15838275 | A | G | 0.023 | 0.689 | 0.107 | EUR | 0.405 | 0.232 |
| rs10833134 | 11 | 19584103 | C | T | 0.141 | 0.050 | 0.922 | AFR | 0.675 | 0.398 |
| rs10741838 | 11 | 20403995 | C | T | 0.154 | 0.933 | 0.485 | EUR | 0.364 | 0.210 |
| rs11029687 | 11 | 26699178 | A | G | 0.118 | 0.663 | 0.024 | EUR | 0.365 | 0.208 |
| rs7927234 | 11 | 27392932 | C | G | 0.012 | 0.110 | 0.852 | AFR | 0.614 | 0.363 |
| rs2993051 | 11 | 29951041 | C | T | 0.070 | 0.775 | 0.820 | NAM | 0.538 | 0.305 |
| rs1396883 | 11 | 34714023 | A | G | 0.023 | 0.559 | 0.029 | EUR | 0.343 | 0.201 |
| rs11034734 | 11 | 38427247 | C | T | 0.019 | 0.512 | 0.010 | EUR | 0.318 | 0.192 |
| rs7937598 | 11 | 44701624 | G | A | 0.118 | 0.819 | 0.228 | EUR | 0.410 | 0.224 |
| rs10437653 | 11 | 46254207 | C | A | 0.019 | 0.563 | 0.937 | NAM | 0.508 | 0.319 |
| rs174570 | 11 | 61353788 | C | T | 0.031 | 0.880 | 0.990 | NAM | 0.799 | 0.502 |
| rs7124676 | 11 | 64069867 | A | G | 0.008 | 0.521 | 0.029 | EUR | 0.318 | 0.191 |
| rs1790740 | 11 | 66886698 | T | C | 0.225 | 0.929 | 0.170 | EUR | 0.542 | 0.308 |
| rs614394 | 11 | 69128449 | G | A | 0.019 | 0.078 | 0.840 | AFR | 0.626 | 0.369 |
| rs1467135 | 11 | 71971690 | T | C | 0.038 | 0.579 | 0.053 | EUR | 0.331 | 0.188 |
| rs7111814 | 11 | 72613473 | C | T | 0.080 | 0.287 | 0.966 | AFR | 0.604 | 0.364 |
| rs568789 | 11 | 83823790 | T | C | 0.076 | 0.242 | 0.995 | AFR | 0.700 | 0.443 |
| rs878874 | 11 | 94228113 | A | G | 0.107 | 0.843 | 0.985 | NAM | 0.628 | 0.374 |
| rs520345 | 11 | 105740221 | T | A | 0.031 | 0.294 | 0.947 | AFR | 0.582 | 0.353 |
| rs12363125 | 11 | 112791126 | T | C | 0.027 | 0.668 | 0.068 | EUR | 0.416 | 0.239 |
| rs10893032 | 11 | 122751364 | A | G | 0.134 | 0.351 | 0.995 | AFR | 0.581 | 0.366 |
| rs485645 | 11 | 125938189 | A | G | 0.046 | 0.641 | 0.966 | NAM | 0.536 | 0.328 |
| rs2510719 | 11 | 126511001 | A | C | 0.034 | 0.574 | 0.039 | EUR | 0.341 | 0.196 |
| rs7103088 | 11 | 129514516 | A | G | 0.019 | 0.111 | 0.917 | AFR | 0.713 | 0.429 |
| rs713279 | 11 | 133068519 | G | T | 0.115 | 0.818 | 0.888 | NAM | 0.542 | 0.305 |
| rs4937920 | 11 | 133970954 | C | T | 0.195 | 0.915 | 0.995 | NAM | 0.576 | 0.343 |
| rs10848644 | 12 | 235550 | T | C | 0.015 | 0.533 | 0.019 | EUR | 0.333 | 0.199 |
| rs10848765 | 12 | 3011756 | C | T | 0.011 | 0.241 | 0.961 | AFR | 0.662 | 0.411 |
| rs7961436 | 12 | 10134132 | G | A | 0.057 | 0.640 | 0.084 | EUR | 0.353 | 0.196 |
| rs11048128 | 12 | 25659380 | G | C | 0.023 | 0.774 | 0.799 | NAM | 0.605 | 0.361 |
| rs2306547 | 12 | 26769152 | T | C | 0.027 | 0.517 | 0.005 | EUR | 0.318 | 0.193 |
| rs1486341 | 12 | 37328330 | C | A | 0.298 | 0.852 | 0.044 | EUR | 0.430 | 0.247 |
| rs2193662 | 12 | 45270637 | T | G | 0.485 | 0.930 | 0.157 | EUR | 0.359 | 0.206 |
| rs10735825 | 12 | 49054606 | T | C | 0.489 | 0.928 | 0.108 | EUR | 0.367 | 0.214 |
| rs10783486 | 12 | 50649053 | G | A | 0.046 | 0.739 | 0.801 | NAM | 0.541 | 0.313 |
| rs4762106 | 12 | 64304740 | A | G | 0.282 | 0.810 | 0.147 | EUR | 0.347 | 0.187 |
| rs893280 | 12 | 65841593 | C | T | 0.004 | 0.255 | 0.927 | AFR | 0.592 | 0.361 |
| rs10492199 | 12 | 66773366 | T | C | 0.050 | 0.687 | 0.980 | NAM | 0.574 | 0.351 |
| rs3759171 | 12 | 70593883 | A | G | 0.034 | 0.732 | 0.015 | EUR | 0.530 | 0.315 |
| rs7971769 | 12 | 78488323 | C | A | 0.126 | 0.221 | 0.951 | AFR | 0.610 | 0.356 |
| rs1795504 | 12 | 79793063 | C | G | 0.317 | 0.865 | 0.191 | EUR | 0.373 | 0.203 |
| rs10466960 | 12 | 81773378 | C | A | 0.023 | 0.086 | 0.850 | AFR | 0.631 | 0.371 |
| rs10732643 | 12 | 87686246 | T | G | 0.011 | 0.070 | 0.879 | AFR | 0.698 | 0.420 |
| rs11113995 | 12 | 107494591 | C | T | 0.309 | 0.924 | 0.364 | EUR | 0.369 | 0.205 |
| rs7958163 | 12 | 112759625 | C | A | 0.034 | 0.702 | 0.172 | EUR | 0.357 | 0.201 |
| rs10774871 | 12 | 115467105 | A | G | 0.244 | 0.912 | 0.330 | EUR | 0.403 | 0.223 |
| rs4767461 | 12 | 115771364 | C | T | 0.107 | 0.279 | 0.971 | AFR | 0.607 | 0.365 |
| rs2694874 | 12 | 124568417 | G | C | 0.038 | 0.734 | 0.211 | EUR | 0.358 | 0.202 |
| rs2585897 | 13 | 20296979 | G | A | 0.076 | 0.857 | 0.884 | NAM | 0.631 | 0.364 |
| rs17359176 | 13 | 22565334 | G | A | 0.204 | 0.941 | 0.995 | NAM | 0.592 | 0.354 |
| rs7990216 | 13 | 24798838 | T | C | 0.271 | 0.898 | 0.083 | EUR | 0.504 | 0.288 |
| rs394307 | 13 | 32467834 | A | G | 0.023 | 0.581 | 0.068 | EUR | 0.329 | 0.189 |
| rs7328696 | 13 | 33109793 | G | A | 0.420 | 0.958 | 0.291 | EUR | 0.396 | 0.228 |
| rs1337973 | 13 | 33759193 | A | G | 0.191 | 0.946 | 0.903 | NAM | 0.543 | 0.309 |
| rs9576996 | 13 | 39813272 | G | C | 0.223 | 0.953 | 0.971 | NAM | 0.564 | 0.329 |
| rs2151236 | 13 | 40667660 | T | A | 0.085 | 0.148 | 0.976 | AFR | 0.741 | 0.451 |
| rs9533623 | 13 | 43278753 | A | G | 0.050 | 0.552 | 0.010 | EUR | 0.325 | 0.192 |
| rs9596011 | 13 | 48266950 | G | A | 0.078 | 0.115 | 0.903 | AFR | 0.649 | 0.375 |
| rs9527650 | 13 | 56860414 | A | G | 0.034 | 0.680 | 0.801 | NAM | 0.519 | 0.304 |
| rs1924381 | 13 | 71219857 | C | T | 0.351 | 0.867 | 0.078 | EUR | 0.397 | 0.225 |
| rs4885162 | 13 | 73767349 | T | C | 0.137 | 0.305 | 0.976 | AFR | 0.580 | 0.349 |
| rs9530435 | 13 | 74891888 | T | C | 0.050 | 0.178 | 0.912 | AFR | 0.623 | 0.364 |
| rs314580 | 13 | 88709705 | A | G | 0.366 | 0.953 | 0.403 | EUR | 0.365 | 0.207 |
| rs9523747 | 13 | 92095202 | A | T | 0.031 | 0.773 | 0.206 | EUR | 0.411 | 0.234 |
| rs2476230 | 13 | 101380444 | G | C | 0.290 | 0.825 | 0.178 | EUR | 0.345 | 0.185 |
| rs10483251 | 14 | 20741117 | G | T | 0.099 | 0.791 | 0.903 | NAM | 0.553 | 0.315 |
| rs1950420 | 14 | 26210860 | C | A | 0.107 | 0.322 | 0.975 | AFR | 0.580 | 0.352 |
| rs7151991 | 14 | 31705323 | G | A | 0.065 | 0.812 | 0.869 | NAM | 0.605 | 0.348 |
| rs1958053 | 14 | 33253458 | T | A | 0.023 | 0.692 | 0.789 | NAM | 0.542 | 0.323 |
| rs1244379 | 14 | 33750102 | C | T | 0.088 | 0.635 | 0.063 | EUR | 0.341 | 0.189 |
| rs1952790 | 14 | 37412427 | T | C | 0.020 | 0.545 | 0.034 | EUR | 0.327 | 0.192 |
| rs11625446 | 14 | 47314308 | C | A | 0.043 | 0.769 | 0.922 | NAM | 0.638 | 0.380 |
| rs1188184 | 14 | 55512165 | G | A | 0.031 | 0.658 | 0.073 | EUR | 0.398 | 0.227 |
| rs928108 | 14 | 56240227 | G | A | 0.080 | 0.732 | 0.898 | NAM | 0.532 | 0.305 |
| rs1889720 | 14 | 56830691 | C | T | 0.008 | 0.072 | 0.853 | AFR | 0.658 | 0.395 |
| rs2296274 | 14 | 60986931 | G | A | 0.073 | 0.198 | 0.961 | AFR | 0.676 | 0.406 |
| rs2251244 | 14 | 61093017 | C | A | 0.717 | 0.181 | 0.985 | EUR | 0.407 | 0.237 |
| rs10145908 | 14 | 62823082 | C | T | 0.011 | 0.086 | 0.830 | AFR | 0.608 | 0.360 |
| rs6573746 | 14 | 66576406 | A | C | 0.512 | 0.056 | 0.743 | EUR | 0.344 | 0.196 |
| rs12435594 | 14 | 68625619 | G | C | 0.073 | 0.705 | 0.937 | NAM | 0.538 | 0.316 |
| rs7142344 | 14 | 78319897 | T | C | 0.031 | 0.141 | 0.976 | AFR | 0.781 | 0.487 |
| rs3825663 | 14 | 89499502 | C | T | 0.058 | 0.056 | 0.854 | AFR | 0.641 | 0.373 |
| rs9323913 | 14 | 94217063 | C | T | 0.050 | 0.693 | 0.866 | NAM | 0.535 | 0.311 |
| rs12434632 | 14 | 100972552 | C | T | 0.145 | 0.794 | 0.976 | NAM | 0.521 | 0.303 |
| rs11160805 | 14 | 103937732 | C | T | 0.088 | 0.814 | 0.981 | NAM | 0.629 | 0.375 |
| rs7146661 | 14 | 104161600 | G | C | 0.084 | 0.729 | 0.145 | EUR | 0.384 | 0.210 |
| rs4983425 | 14 | 105032574 | G | A | 0.054 | 0.050 | 0.820 | AFR | 0.600 | 0.347 |
| rs7164838 | 15 | 32754866 | G | A | 0.080 | 0.702 | 0.976 | NAM | 0.536 | 0.320 |
| rs8041147 | 15 | 35085650 | T | C | 0.092 | 0.902 | 0.223 | EUR | 0.546 | 0.311 |
| rs2304580 | 15 | 38816959 | T | C | 0.136 | 0.842 | 0.927 | NAM | 0.555 | 0.315 |
| rs1678986 | 15 | 40308480 | C | G | 0.148 | 0.838 | 0.309 | EUR | 0.362 | 0.197 |
| rs8030587 | 15 | 40769098 | A | G | 0.015 | 0.197 | 0.966 | AFR | 0.713 | 0.443 |
| rs735480 | 15 | 42939663 | T | C | 0.317 | 0.894 | 0.015 | EUR | 0.482 | 0.287 |
| rs1153858 | 15 | 43439995 | C | T | 0.023 | 0.735 | 0.136 | EUR | 0.430 | 0.246 |
| rs1453858 | 15 | 46091402 | T | A | 0.206 | 0.911 | 0.228 | EUR | 0.489 | 0.273 |
| rs728244 | 15 | 53604277 | A | G | 0.057 | 0.151 | 0.932 | AFR | 0.678 | 0.400 |
| rs474875 | 15 | 56873995 | A | C | 0.046 | 0.717 | 0.189 | EUR | 0.354 | 0.198 |
| rs7176730 | 15 | 58364666 | C | T | 0.019 | 0.580 | 0.879 | NAM | 0.504 | 0.310 |
| rs2415047 | 15 | 67675432 | A | C | 0.351 | 0.896 | 0.131 | EUR | 0.418 | 0.235 |
| rs746655 | 15 | 71894730 | A | G | 0.004 | 0.122 | 0.854 | AFR | 0.608 | 0.363 |
| rs901130 | 15 | 72360960 | C | G | 0.134 | 0.697 | 0.039 | EUR | 0.382 | 0.215 |
| rs2627316 | 15 | 78829871 | G | A | 0.027 | 0.532 | 0.024 | EUR | 0.319 | 0.187 |
| rs7178655 | 15 | 82555245 | G | T | 0.092 | 0.722 | 0.102 | EUR | 0.404 | 0.222 |
| rs11631761 | 15 | 90102302 | A | G | 0.034 | 0.614 | 0.898 | NAM | 0.512 | 0.308 |
| rs11074130 | 15 | 91384746 | G | T | 0.321 | 0.922 | 0.233 | EUR | 0.431 | 0.240 |
| rs4402506 | 15 | 91931362 | C | T | 0.050 | 0.588 | 0.015 | EUR | 0.357 | 0.209 |
| rs7173885 | 15 | 93608135 | G | C | 0.008 | 0.249 | 0.917 | AFR | 0.581 | 0.351 |
| rs2301426 | 16 | 651906 | T | C | 0.038 | 0.670 | 0.087 | EUR | 0.393 | 0.222 |
| rs1011489 | 16 | 5607394 | T | C | 0.115 | 0.679 | 0.010 | EUR | 0.391 | 0.228 |
| rs4787040 | 16 | 7500981 | T | A | 0.080 | 0.714 | 0.039 | EUR | 0.448 | 0.255 |
| rs9937557 | 16 | 8244391 | C | G | 0.202 | 0.940 | 0.927 | NAM | 0.545 | 0.310 |
| rs12929500 | 16 | 10600333 | A | C | 0.134 | 0.848 | 0.177 | EUR | 0.478 | 0.264 |
| rs7185307 | 16 | 11954400 | T | C | 0.088 | 0.751 | 0.121 | EUR | 0.426 | 0.234 |
| rs9923547 | 16 | 12685472 | A | C | 0.011 | 0.258 | 0.942 | AFR | 0.612 | 0.374 |
| rs30237 | 16 | 14282146 | G | A | 0.084 | 0.162 | 0.903 | AFR | 0.604 | 0.346 |
| rs4347621 | 16 | 50241402 | G | A | 0.065 | 0.152 | 0.903 | AFR | 0.625 | 0.361 |
| rs1787781 | 16 | 55898649 | A | G | 0.015 | 0.163 | 0.879 | AFR | 0.599 | 0.355 |
| rs8048832 | 16 | 71246151 | T | C | 0.153 | 0.882 | 0.320 | EUR | 0.411 | 0.227 |
| rs10871351 | 16 | 77080407 | G | A | 0.019 | 0.230 | 0.937 | AFR | 0.628 | 0.380 |
| rs8043824 | 16 | 78404643 | G | A | 0.206 | 0.253 | 0.985 | AFR | 0.598 | 0.362 |
| rs1452501 | 16 | 79180763 | C | T | 0.214 | 0.942 | 0.980 | NAM | 0.572 | 0.336 |
| rs2967340 | 16 | 80815052 | A | G | 0.225 | 0.848 | 0.092 | EUR | 0.465 | 0.259 |
| rs2086824 | 16 | 87998747 | C | A | 0.038 | 0.565 | 0.015 | EUR | 0.346 | 0.204 |
| rs4791868 | 17 | 9638969 | G | C | 0.141 | 0.937 | 0.126 | EUR | 0.648 | 0.376 |
| rs8068853 | 17 | 19342689 | T | C | 0.092 | 0.716 | 0.034 | EUR | 0.444 | 0.253 |
| rs8081410 | 17 | 33096391 | G | A | 0.130 | 0.691 | 0.093 | EUR | 0.348 | 0.189 |
| rs2197159 | 17 | 44832634 | T | C | 0.027 | 0.699 | 0.828 | NAM | 0.560 | 0.333 |
| rs7211426 | 17 | 51009547 | A | G | 0.092 | 0.212 | 0.951 | AFR | 0.637 | 0.376 |
| rs1426741 | 17 | 51138511 | G | T | 0.317 | 0.867 | 0.121 | EUR | 0.406 | 0.225 |
| rs7211655 | 17 | 53210594 | A | G | 0.099 | 0.711 | 0.976 | NAM | 0.514 | 0.304 |
| rs1197062 | 17 | 55995900 | C | A | 0.008 | 0.055 | 0.873 | AFR | 0.706 | 0.428 |
| rs2052074 | 17 | 56516428 | C | T | 0.118 | 0.177 | 0.966 | AFR | 0.677 | 0.403 |
| rs4790939 | 17 | 60853432 | G | C | 0.107 | 0.115 | 0.908 | AFR | 0.635 | 0.365 |
| rs918077 | 17 | 67676973 | T | C | 0.321 | 0.880 | 0.248 | EUR | 0.364 | 0.197 |
| rs4789193 | 17 | 70916391 | A | T | 0.065 | 0.191 | 0.951 | AFR | 0.670 | 0.400 |
| rs680173 | 18 | 2864364 | C | G | 0.038 | 0.686 | 0.820 | NAM | 0.527 | 0.309 |
| rs9948473 | 18 | 5735074 | A | G | 0.252 | 0.837 | 0.248 | EUR | 0.347 | 0.186 |
| rs17519184 | 18 | 6976714 | G | A | 0.042 | 0.677 | 0.887 | NAM | 0.546 | 0.323 |
| rs9945476 | 18 | 9776265 | A | G | 0.050 | 0.816 | 0.272 | EUR | 0.403 | 0.228 |
| rs628419 | 18 | 12003384 | C | T | 0.112 | 0.641 | 0.005 | EUR | 0.354 | 0.208 |
| rs507163 | 18 | 32161626 | T | C | 0.118 | 0.751 | 0.155 | EUR | 0.381 | 0.206 |
| rs12185395 | 18 | 38764116 | T | C | 0.019 | 0.579 | 0.034 | EUR | 0.360 | 0.211 |
| rs1893441 | 18 | 45612749 | A | G | 0.080 | 0.805 | 0.966 | NAM | 0.627 | 0.372 |
| rs11667393 | 19 | 54353839 | A | G | 0.069 | 0.738 | 0.199 | EUR | 0.360 | 0.198 |
| rs6112672 | 20 | 1994772 | G | T | 0.057 | 0.837 | 0.209 | EUR | 0.481 | 0.272 |
| rs6056505 | 20 | 9191782 | C | T | 0.328 | 0.928 | 0.388 | EUR | 0.353 | 0.196 |
| rs2889678 | 20 | 30653654 | T | C | 0.031 | 0.703 | 0.058 | EUR | 0.462 | 0.266 |
| rs4911320 | 20 | 31377692 | T | G | 0.443 | 0.056 | 0.845 | EUR | 0.325 | 0.191 |
| rs6088466 | 20 | 32377195 | G | A | 0.034 | 0.696 | 0.981 | NAM | 0.609 | 0.378 |
| rs221308 | 20 | 34709812 | A | G | 0.050 | 0.804 | 0.879 | NAM | 0.631 | 0.369 |
| rs1934915 | 20 | 36329274 | A | G | 0.141 | 0.852 | 0.942 | NAM | 0.564 | 0.322 |
| rs4812381 | 20 | 37420974 | A | G | 0.046 | 0.694 | 0.845 | NAM | 0.532 | 0.310 |
| rs6101991 | 20 | 38515722 | G | A | 0.038 | 0.104 | 0.853 | AFR | 0.608 | 0.352 |
| rs4812831 | 20 | 42451674 | G | A | 0.168 | 0.904 | 0.961 | NAM | 0.586 | 0.338 |
| rs1201686 | 20 | 45044330 | G | C | 0.237 | 0.903 | 0.403 | EUR | 0.349 | 0.192 |
| rs731803 | 20 | 53941220 | C | T | 0.019 | 0.602 | 0.820 | NAM | 0.499 | 0.302 |
| rs1877751 | 20 | 57401301 | A | G | 0.034 | 0.669 | 0.869 | NAM | 0.544 | 0.323 |
| rs2242865 | 21 | 15948902 | G | T | 0.011 | 0.156 | 0.932 | AFR | 0.695 | 0.422 |
| rs2236611 | 21 | 32607151 | T | C | 0.198 | 0.741 | 0.053 | EUR | 0.372 | 0.207 |
| rs2300395 | 21 | 35167068 | T | C | 0.011 | 0.294 | 0.951 | AFR | 0.594 | 0.366 |
| rs2835367 | 21 | 36805244 | T | C | 0.256 | 0.899 | 0.175 | EUR | 0.471 | 0.262 |
| rs2836181 | 21 | 38477680 | T | C | 0.016 | 0.049 | 0.801 | AFR | 0.604 | 0.358 |
| rs2837352 | 21 | 40285648 | C | T | 0.053 | 0.879 | 0.927 | NAM | 0.721 | 0.429 |
| rs12329755 | 21 | 41518707 | C | G | 0.019 | 0.390 | 0.990 | AFR | 0.576 | 0.375 |
| rs2187239 | 21 | 42293624 | A | C | 0.023 | 0.684 | 0.819 | NAM | 0.550 | 0.329 |
| rs762421 | 21 | 44439989 | A | G | 0.038 | 0.634 | 0.937 | NAM | 0.535 | 0.325 |
| rs5748014 | 22 | 17528721 | C | A | 0.057 | 0.752 | 0.233 | EUR | 0.354 | 0.197 |
| rs5754506 | 22 | 32013735 | C | T | 0.008 | 0.135 | 0.858 | AFR | 0.599 | 0.356 |
| rs132663 | 22 | 34892970 | A | T | 0.027 | 0.684 | 0.893 | NAM | 0.581 | 0.351 |
| rs5757362 | 22 | 37636026 | C | T | 0.065 | 0.647 | 0.985 | NAM | 0.514 | 0.313 |
| rs470113 | 22 | 39059560 | A | G | 0.065 | 0.835 | 0.858 | NAM | 0.615 | 0.354 |
| rs9611566 | 22 | 40098571 | C | T | 0.061 | 0.781 | 0.966 | NAM | 0.637 | 0.382 |
| rs16990991 | 22 | 42499017 | G | A | 0.126 | 0.778 | 0.985 | NAM | 0.536 | 0.316 |
| rs1557553 | 22 | 43139648 | C | T | 0.086 | 0.892 | 0.947 | NAM | 0.690 | 0.406 |
| rs801712 | 22 | 45468907 | C | G | 0.126 | 0.805 | 0.223 | EUR | 0.392 | 0.213 |
