# Supplementary material for: Development of a Panel of Genome-Wide Ancestry Informative Markers to Study Admixture Throughout the Americas
Source: PLoS Genet. 2012 Mar 8;8(3):e1002554. doi: 10.1371/journal.pgen.1002554 (PMC3297575; doi:10.1371/journal.pgen.1002554)
Supplement: Text S1 — Supplemental methods, including detailed ethics statement and description of samples obtained for genotyping of populations throughout the Americas. (DOCX) [file pgen.1002554.s004.docx]

Development of a Panel of Genome-wide Ancestry Informative Markers to Study Admixture Throughout the Americas

Joshua Mark Galanter*,^1^ Juan Carlos Fernandez,^2^ Christopher R. Gignoux,^1^ Jill Barnholtz-Sloan,^3^ Ceres Fernandez,^4^ Marc Via,^5^ Alfredo Hidalgo-Miranda,^2^ Alejandra V. Contreras,^2^ Laura Uribe Figueroa,^2^ Paola Raska,^2^ Gerardo Jimenez-Sanchez,^2^ Irma Silva Zolezzi,^2^ Maria Torres,^4^ Clara Ruiz Ponte,^4^ Yarimar Ruiz,^4^ Antonio Salas,^4^ Elizabeth Nguyen,^1^ Celeste Eng,^1^ Lisbeth Borjas,^6^ William Zabala,^4,6^ Guillermo Barreto,^7^ Fernando Rondón,^7^ Adriana Ibarra,^8^ Patricia Taboada,^4,9^ Liliana Porras,^4,10^ Fabián Moreno,^11^ Abbigail Bigham,^12^ Gerardo Gutierrez,^13^ Tom Brutsaert,^14^ Fabiola León-Velarde,^15^ Lorna G. Moore,^16^ Enrique Vargas,^17^ Miguel Cruz,^18^ Jorge Escobedo,^19^ Jose Rodriguez-Santana,^20^ William Rodriguez-Cintrón,^21^ Rocio Chapela,^22^ Jean G. Ford,^23^ Carlos Bustamante,^24^ Daniela Seminara,^25^ Mark Shriver,^26^ Elad Ziv,^1^ Esteban Gonzalez Burchard,^1^ Robert Haile,^27^ Esteban Parra,^28, 29^ Angel Carracedo^4, 29^ for the LACE consortium.

1. University of California, San Francisco; San Francisco, CA
2. Instituto Nacional de Medicina Genómica; Mexico City, DF, Mexico
3. Case Western Reserve University, Cleveland, OH
4. Fundación Pública Galega de Medicina Xenómica (SERGAS)-CIBERER, Universidade de Santiago de Compostela, Santiago de Compostela, Spain
5. Universitat de Barcelona, Barcelona, Spain
6. Universidad del Zulia, Maracaibo, Zulia, Venezuela
7. Universidad del Valle, Santiago de Cali, Colombia
8. Universidad de Antioquia, Medellín, Antioquia, Colombia
9. Instituto de Investigaciones Forenses, Sucre, Bolivia
10. Universidad Tecnológica de Pereira, Pereira, Colombia
11. Unidad de Genética forense, Servicio Médico-legal de Chile, Santiago de Chile, Chile
12. University of Michigan, Ann Arbor, MI
13. University of Colorado at Boulder, Boulder, CO
14. Syracuse University, Syracuse, NY
15. Universidad Peruana Cayetano Heredia, Lima, Peru
16. Wake Forest University, Winston-Salem, NC
17. Universidad Mayor de San Andrés, La Paz, Bolivia
18. Centro Medico Nacional Siglo XXI, IMSS, Mexico City, Mexico
19. Hospital General Regional 1, Mexico City, Mexico
20. Centro de Neumología Pediátrica, San Juan, PR
21. VA Caribbean Health System, San Juan, PR
22. Instituto Nacional de Enfermedades Respiratorias (INER), Mexico City, Mexico
23. Johns Hopkins Bloomberg School of Public Health, Baltimore, MD
24. Stanford University, Stanford, CA
25. National Cancer Institute, Bethesda, MD
26. Penn State University, University Park, PA
27. University of Southern California, Los Angeles, CA
28. University of Toronto at Mississauga, Mississauga, Ontario, Canada
29. These authors contributed equally to this manuscript

* Address correspondence to:

Joshua Galanter

UCSF Box 2911

San Francisco, CA 94143

E-mail: joshua.galanter@ucsf.edu

# Supplemental Methods

## Ethics and informed consent statement

Samples for the selection of AIMs were collected with the input from local communities and approval of local ethics committees. Informed consent from participants allowing research into human variation was obtained. In addition to local ethical review boards, the Institutional Review Board at The Pennsylvania State University approved sample collection for the Nahua, Quechua, and Aymara populations and the National Institute of Genomic Medicine (INMEGEN) approved sample collection for the Zapoteca, Tepehuano, and Maya samples. To prevent loss of privacy through individual identification through public release of genotypes, the genotypes of Native American ancestral populations provided are permuted.

Samples for validation were collected with informed consent from all participants allowing collection of DNA and genotyping. The studies were approved by the local institutional review boards, the Committee on Human Research at UCSF (for the GALA Mexican and Puerto Rican samples), the Ethics Review Office at the University of Toronto and the Institutional Review Board of the Medical Center “Siglo XXI” in Mexico (for the Mexico City samples), and the Institutional Review Board at the Mexican National Institute of Genomic Medicine (for the MGDP-INMEGEN samples).

Samples collected for the third phase of the study, examining variation throughout Latin America, were collected with the approval of local ethics committees and informed consent from all participants for research into human variation. The Ethical Committee of the University of Santiago de Compostela (Spain) approved this study.

# Genotyping of populations throughout Latin America

**Bolivia**

The Bolivian samples were collected from four departments: Beni, Cochabamba, La Paz, and Yungas.

Beni Department: Samples from 10 indigenous and mestizo individuals were collected in the Los Llanos (tropical plains, altitude 236 m above sea level) region in Northern Bolivia.

Cochabamba Department: Samples from 12 indigenous and mestizo individuals were collected in the Los Valles (valley) region in central Bolivia.

La Paz department: Samples from 38 subjects were collected from two distinct regions within this department. 27 individuals were recruited from the subtropical Yungas region in Western Bolivia (altitude of 1700 m). These subjects came from the small community of Tocaña (n = 18), and the town of Coroico (n = 9). The Yungas region is characterized by the presence of indigenous populations and scattered Afro-Bolivian communities. The remaining 11 subjects were indigenous and Mestizo individuals from the Altiplano (high plains, altitude 3500 m) region, recruited in rural Copacabana and urban La Paz.

**Colombia**

Four samples (three indigenous populations, one Mestizo population, and two Afro-Colombian population were recruited. Colombian mestizos (n = 19) were recruited in the Northwest region of Antioquia. Afro-Colombians were recruited fin Western Colombia from Mulaló (n = 28) and Chocó (n = 35). Indigenous Coyaima (n = 19) were recruited from central Colombia. Indigenous Pastos (n = 26) and Awa (n = 22) were recruited from southern Colombia. These two populations live in geographically isolated plateaus at an altitude greater than 2000 meters.

**Argentina**

From Argentina, we recruited 14 Wichí (also known as “Mataco”) individuals. This indigenous population inhabits territories around the headwaters of the Bermejo and Pilcomayo Rivers, in the province of Chaco in northern Argentina.

**Venezuela**

Samples from one mestizo and four indigenous and populations were collected in Venezuela.

Indigenous Warao (n = 20) were recruited in Isla Tres Caños (Delta Amacuro State in Eastern Venezuela). The Warao live in artificial islands built with the sediments brought by the Orinoco River and speak the isolated language Warao. Twnety indigenous Panare (also known as E´ñepa) were recruited in Santa María de Uonquen (Bolivar Province in Southeastern Venezuela). The Panare occupy a vast territory of about 20,000 km^2^ located in the northwestern part of Bolivar Province, with a small enclave in the province of Amazonas. They speak Panare (from the Carib language family). Twenty indigenous Pemon were recruited in Maiapure in Bolivar Province. The Pemon live in the Gran Sabana region and Canaima National Park near the border with Brazil and Guyana. They speak Pemon, one of the languages of the Carib family. Samples from indigenous Wayu (n = 20) were recruited from a geographically isolated area in the desert of La Guajira between Colombia and Venezuela in the Northwest of the country.

Mestizo individuals (n = 20) were recruited from Maracaibo, Venezuela’s second largest city, located in the northwest part of the country.

**Chile**

From Chile, random samples were collected from unrelated mestizo individuals living in the Northern (n = 20) and Southern (n = 20) areas of the country.
